# Selective brain regional changes in lipid profile with human aging

**DOI:** 10.1007/s11357-022-00527-1

**Published:** 2022-02-11

**Authors:** Natalia Mota-Martorell, Pol Andrés-Benito, Meritxell Martín-Gari, José Daniel Galo-Licona, Joaquim Sol, Anna Fernández-Bernal, Manuel Portero-Otín, Isidro Ferrer, Mariona Jove, Reinald Pamplona

**Affiliations:** 1 Department of Experimental Medicine, University of Lleida—Lleida Biomedical Research Institute (UdL-IRBLleida), 25198 Lleida, Spain; 2 Center for Biomedical Research On Neurodegenerative Diseases (CIBERNED), Institute of Health Carlos III, 28220 Madrid, Spain; 3 Department of Pathology and Experimental Therapeutics, University of Barcelona, L’Hospitalet de Llobregat, 08907 Barcelona, Spain; 4 Institute of Biomedical Research of Bellvitge (IDIBELL), 08907 Hospitalet de Llobregat, Spain

**Keywords:** Average chain length, Cerebral cortex, Human brain regions, Fatty acid profile, Peroxidizability index, Polyunsaturated fatty acids

## Abstract

Fatty acids are key components in the structural diversity of lipids and play a strategic role in the functional properties of lipids which determine the integrity of neuronal and glial cell membranes, the generation of lipid signaling mediators, and the chemical reactivity of acyl chains. The present study analyzes using gas chromatography the fatty acid profiles of 13 regions of the human central nervous system in healthy individuals ranging from 40 to 80 years old. The outcomes suggest the existence of general traits in fatty acid composition such as an average chain length of 18 carbon atoms, high monounsaturated fatty acid content, and predominance in polyunsaturated fatty acids of those of series n-6 over series n-3 which are shared by all brain regions regardless of age. Our results also show a general sustained and relatively well-preserved lipid profile throughout the adult lifespan in most studied regions (olive, upper vermis, substantia nigra, thalamus, hippocampus, putamen, caudate, occipital cortex, parietal cortex, entorhinal cortex, and frontal cortex) with minor changes that are region-dependent. In contrast, of particular relevance is the involvement of the inferior temporal cortex and cingulate cortex. It is proposed that during normal human brain aging, the lipid profile is resistant to changes with age in most human brain regions to ensure cell survival and function, but some particular regions involved in specific memory domains are greatly affected.

**Supplementary Information:**

The online version contains supplementary material available at 10.1007/s11357-022-00527-1.

## Introduction

Human brain evolution and lipids are closely linked [1, 2]. Structural and functional diversity and abundance of lipids are also traits of the human brain [1, 3–5]. As result, all lipid classes are present in the nervous system, likely as expression and support of the structural and functional complexity of the system [4, 6]. This abundance and diversity of lipids require a quarter of the total brain energy to maintain cellular activity involved in de novo lipid biosynthesis, remodeling, turnover, and synthesis of lipid-derived mediators, as well as continuous adjustment of the spatial and temporal lipid organization of cell membranes [7, 8].

Fatty acids are major components of glycerolipids, glycerophospholipids, and sphingolipids. The combination of fatty acids or fatty acids with different head groups (depending on the lipid class) can generate around 10,000 different lipid molecular species [6]. The length (number of carbon atoms) of the acyl chain and number of double bonds are determinants of the geometric traits of lipids influencing membrane organization and function [4]. Furthermore, fatty acids are substrates for the generation of lipid signaling mediators [9]. An additional trait assigned to fatty acids is their chemical reactivity in the face of oxidative conditions [10], which, by extension, determines the susceptibility to oxidative stress for a given membrane [10]. Thus, polyunsaturated side chains are much more easily attacked by oxidant agents than saturated or monounsaturated fatty acid side chains.

In this context, it has been suggested that the morphological and functional diversity among neural cells in the human brain is also achieved by the expression of region-specific lipid profiles [10]. In agreement with this hypothesis, we recently demonstrated that particular fatty acids are significant discriminators among human brain regions and that these specific fatty acid profiles generate a differential cross-regional selective neural vulnerability (expressed by the double bond index and peroxidizability index). In other words, there is a region-specific vulnerability to lipid peroxidation in human brain [10]. Changes in global lipid composition have been reported in human brain aging, and in several age-associated neurodegenerative diseases such as Alzheimer disease [5]. However, little is known about alterations occurring across brain regions throughout the adult human lifespan.

The present study analyzes the fatty acid profiles of total lipids from the gray matter of 13 different regions of the human central nervous system belonging to the hindbrain (*olive* and *upper vermis*), midbrain (*substantia nigra*), diencephalon (*thalamus*), subcortical telencephalon (*hippocampus*, *head of the caudate*, and *anterior putamen*), and cortical telencephalon (*occipital cortex areas 17–18*, *parietal cortex area 7*, *inferior temporal cortex area 20*, *entorhinal cortex*, *frontal cortex area 8*, and *cingulate gyrus area 24*), in healthy individuals without co-morbidities ranging from 40 to 80 years old. The brain areas were selected on the basis of their selective vulnerability to neurodegenerative diseases in aging. Our results show selective brain regional changes in lipid profile with human healthy aging.

## Material and methods

### Chemicals

Unless otherwise specified, all reagents were from Sigma-Aldrich and were of the highest purity available.

### Human samples

Brain samples were obtained from the Institute of Neuropathology Brain Bank, a branch of the HUB-ICO-IDIBELL Biobank, following the guidelines of Spanish legislation (Real Decreto 1716/2011) and the approval of the local ethics committee (CEIC/1981).

At autopsy, one hemisphere was fixed in 4% buffered formalin for about 3 weeks while the other hemisphere was cut in coronal Sects. 0.5-cm thick; selected areas of the brain were dissected, immediately frozen on metal plates over dry ice, placed in labelled plastic bags, and stored at – 80 °C until use. Procedures were designed to preserve post-mortem material under optimal conditions for morphological and biochemical studies [11]. The post-mortem delay ranged from 2 to 14 h 40 min (see Table 1).

**Table 1:** Summary of cases examined

Case	Gender	Age (years)	Post-mortem delay	Neuropathology	Cause of death
1	Male	40	5 h 10 min	NL	CA
2	Female	40	8 h 45 min	NL	PNEU
3	Male	44	6 h 40 min	NL	THR-EMB
4	Male	45	4 h 5 min	CRIB	C-INF
5	Female	46	7 h 15 min	CRIB	MYO
6	Female	48	4 h 5 min	NL	CA
7	Male	52	9 h 30 min	NL	PNEU
8	Male	52	4 h 40 min	NL	C-INF
9	Male	57	5 h 20 min	NL	PNEU
10	Male	61	4 h 30 min	I-II	CA
11	Male	66	6 h 25 min	I-II	THR-EMB
12	Male	67	14 h 40 min	I-II	CA
13	Male	70	2 h 00 min	I-II	CA
14	Female	75	6 h 10 min	I-II + CRIB	C-INF
15	Male	76	6 h 30 min	I-II	PNEU
16	Male	77	6 h 55 min	I-II + CRIB	C-INF
17	Female	79	6 h 25 min	I-II + CRIB	INT-INF

*NL*, no lesions; *CRIB*, status cribosus; *I-II*, neurofibrillary tangle pathology stages I–II of Braak and Braak; *CA*, carcinoma; *PNEU*, pneumonia; *C-INF*, cardiac infarction; *THR-EMB*, pulmonary thrombosis-embolism; *MYO*, myocardiopathy; *INT-INF*, intestinal infarction

The neuropathological study, to discriminate healthy brains, was carried out in every case as previously described [11]. Briefly, the neuropathological study was carried out on formalin-fixed, paraffin-embedded samples of 26 brain regions. De-waxed sections, 4-µm thick, were stained with hematoxylin and eosin, and Klüver Barrera, or processed for immunohistochemistry to β-amyloid, phosphorylated tau (including clone AT8), α-synuclein, ubiquitin, p62, TDP43, glial fibrillary protein, and microglia markers.

Adult and middle-aged individuals (< 60 years) had no clinical or neuropathological alterations, whereas old-aged individuals (> 60 years) had no clinical symptoms and neuropathological alteration restricted to stage I of neurofibrillary degeneration. Since the majority of human beings aged 65 years have stages I–II of neurofibrillary tangle pathology [12, 13], the old-aged group was considered representative of normal brain aging. β-Amyloid deposition was absent in every case. All cases included in this study were without co-morbidities. Samples were from individuals with no neurological symptoms and without systemic and focal infectious or inflammatory and autoimmune diseases. Cases with disseminated malignant diseases, metabolic syndrome, or drug abuse (for instance, excessive ethanol consumption) were not included. Special care was also taken to not include cases with prolonged agonal state (patients subjected to intensive care or experiencing hypoxia). After neuropathological examination, cases with both neurodegenerative and vascular diseases were excluded excepting those with stages I–II of neurofibrillary tangle pathology. Finally, cases with associated neurodegenerative processes (i.e., TDP-43 proteinopathy, argyrophilic grain pathology, α-synucleinopathy, and other tauopathies) were also excluded.

Following initial screening, the present series included 17 cases: 12 males and 5 females, with age ranging from 40 to 80 years. Table 1 summarizes cases examined in the present series. The gray matter from cerebral cortex (frontal area 8, parietal area 7, inferior temporal area 20, occipital areas 17–18, cingulate gyrus area 24, entorhinal cortex, and hippocampus), striatum (head of the caudate and anterior putamen), thalamus, substantia nigra, upper vermis, and olive were dissected and used for biochemical studies.

### Fatty acid profile

Fatty acyl groups of total lipids from gray matter of 13 human brain regions were analyzed as methyl ester derivatives (FAMEs) by gas chromatography (GC) as previously described [10, 14]. Firstly, 50 mg of tissue samples was homogenized in a buffer containing 180 mM KCl, 5 mM MOPS, 2 mM EDTA, 1 mM diethylenetriaminepentaacetic acid, and 1 μM butylated hydroxytoluene. Tissue samples were randomized prior to lipid extraction. Quality control samples were included at a ratio of 1:10. Then, total lipids from human brain homogenates were extracted with chloroform:methanol 2:1 (v/v). The chloroform phase was separated and evaporated under N_2_, and the fatty acyl groups were transesterified by incubation in 2.5 ml of 5% methanolic HCl at 75 °C for 90 min. The resulting fatty acid methyl esters were extracted by adding 1 ml of saturated NaCl solution and 2.5 ml of *n*-pentane. Finally, the n-pentane phase was separated, evaporated under N_2_, and redissolved in 75 µl of carbon disulfide. Two microliters was used for GC analysis.

Separation was performed with a DBWAX capillary column (30 m × 0.25 mm × 0.25 μm) in a GC System 7890A with a Series Injector 7683B and a FID detector (Agilent Technologies, Barcelona, Spain). Sample injection was in the splitless mode. The injection port was maintained at 250 °C, and the detector at 250 °C. The temperature program was 5 min at 145 °C, then 2 °C/min to 245 °C, and finally hold at 245 °C for 10 min, with a post-run at 250 °C for 10 min. So, total run time was 65 min, with a post-run time of 10 min. Identification of fatty acid methyl esters was made by comparison with authentic standards (Larodan Fine Chemicals, Malmö, Sweden) using a specific software of data analysis for GC from Agilent (OpenLAB CDS ChemStation v. C.01.10; Agilent Technologies, Barcelona, Spain) and subsequent revision and confirmation by an expert. Results are expressed as mol%.

The following fatty acyl indices were also calculated: saturated fatty acids (SFA); unsaturated fatty acids (UFA); ratio SFA/UFA; monounsaturated fatty acids (MUFA); polyunsaturated fatty acids (PUFA) from n-3 and n-6 series (PUFAn-3 and PUFAn-6); and average chain length (ACL) = [(Σ% Total14 × 14) + (Σ% Total16 × 16) + (Σ% Total18 × 18) + (Σ% Total20 × 20) + (Σ% Total22 × 22) + (Σ% Total24 × 24)]/100. The density of double bonds in the membrane was calculated with the double bond index, DBI = [(1 × Σmol% monoenoic) + (2 × Σmol% dienoic) + (3 × Σmol% trienoic) + (4 × Σmol% tetraenoic) + (5 × Σmol% pentaenoic) + (6 × Σmol% hexaenoic)]. Membrane susceptibility to peroxidation was calculated with the peroxidizability index, PI = [(0.025 × Σmol% monoenoic) + (1 × Σmol% dienoic) + (2 × Σmol% trienoic) + (4 × Σmol% tetraenoic) + (6 × Σmol% pentaenoic) + (8 × Σmol% hexaenoic)].

Elongase and desaturase activity was estimated from specific product/substrate ratios. For desaturase activity: D9D (n-7) = 16:1n-7/16:0; D9D (n-9) = 18:1n-9/18:0; D5D (n-6) = 20:4n-6/20:3n-6; D6D (n-3) (a) = 18:4n-3/18:3n-3; D6D (n-3) (b) = 24:6n-3/24:5n-3. For elongase activity: Elovl3 (n-9) (a) = 20:1n-9/18:1n-9; Elovl3 (n-9) (b) = 22:1n-9/20:1n-9; Elovl3 (n-9) (c) = 24:1n-9/22:1n-9;Elovl6 = 18:0/16:0; Elovl1-3–7 (a) = 20:0/18:0; Elovl1-3–7 (b) = 22:0/20:0; Elovl1-3–7 (c) = 24:0/22:0; Elovl 5(n-6) = 20:2n-6/18:2n-6; Elovl2-5 (n-6) = 22:4n-6/20:4n-6; Elovl 2–5(n-3) = 22:5n-3/20:5n-3, and Elovl 2(n-3) = 24:5n-3/22:5n-3. Finally, peroxisomal β-oxidation (PβOx) was estimated according to the ratio 22:6n-3/24:6n-3.

### Statistics

Data were expressed as mean values ± SEM. Student’s *t*-test was used to evaluate differences between age groups. Spearman’s rank correlation was employed to assess possible relations for variables with age. Statistical significance was adjusted for multiple testing by controlling the false discovery rate according to the Benjamini–Hochberg method using a maximum discovery rate of 10%. Graphs were made and *t*-tests performed using GraphPad prism 8.0.1. Spearman correlation was made using IBM SPSS Statistics (v24.0.0.0). Spearman correlation matrix was constructed using RStudio (v1.3.1073). Functions used were included in the packages *corrplot* [15] and *Hmisc* [16]. *p* values inferior to 0.05 were considered statistically significant.

## Results

### General traits shared by human brain regions

The changes in brain fatty acid profile along the aging process were assessed by analyzing 25 different fatty acid species across 13 different brain regions, including the olive (medulla oblongata) and vermis (cerebellum) (hindbrain); substantia nigra (midbrain); thalamus (diencephalon); hippocampus, caudate, and putamen (subcortical telencephalon); and occipital, parietal, temporal, entorhinal, frontal, and cingulate cortex (cortical telencephalon). Results revealed that 16:0, 18:0, and 18:1n-9 are the most abundant fatty acids in human healthy brain, in both the middle-aged and the elderly, accounting for approximately 60% of global fatty acid composition (Table 2). In addition, specific enrichment of 20:4n-6 and 22:6n-3 is found in all regions. As a result, the average chain length (ACL) is maintained at around 18 carbon atoms, with a predominant presence of monounsaturated fatty acids (MUFA), followed by polyunsaturated fatty acids (PUFA), showing a higher relative abundance of PUFAn-6 with respect to PUFAn-3.

**Table 2:** Fatty acid composition of total lipids from different human brain regions comparing middle-aged and elderly individuals. Healthy individuals were grouped as middle-aged (< 60 years) and elderly (> 60 years). Differences between groups were assessed by applying a *t*-test and corrected by FDR method of Benjamini and Hochberg, with *Q* = 10%. Values are reported in mol% as mean ± SEM from 8 to 9 individuals × group. *P* < 0.05 was selected as the minimum level of statistical significance. Significance compared to middle-aged group prior FDR correction is denoted using asterisks (*P < 0.05; **P < 0.01; ***P < *0.001)*

		HINDBRAIN																MIDBRAIN					
		Olive									Vermis							Substantia nigra					
		Middle-aged				Elderly			Sig		Middle-aged		Elderly			Sig		Middle-aged		Elderly			Sig
14:0		1.84 ± 0.19				1.29 ± 0.12*			0.546		1.08 ± 0.05		0.99 ± 0.05			0.803		0.61 ± 0.02		0.65 ± 0.02			0.578
16:0		15.36 ± 0.26				15.78 ± 0.44			0.775		24.5 ± 0.56		24.7 ± 0.38			0.908		17.55 ± 0.18		18.28 ± 0.49			0.457
16:1n7		2.23 ± 0.09				2.11 ± 0.25			0.775		1.22 ± 0.08		1.14 ± 0.07			0.803		1.22 ± 0.03		1.35 ± 0.05*			0.229
18:0		20.11 ± 0.52				19.24 ± 0.81			0.597		20.28 ± 0.18		20.82 ± 0.48			0.803		22.57 ± 0.13		22.98 ± 0.29			0.571
18:1n9		30.57 ± 0.35				31.3 ± 2.48			0.597		20.81 ± 0.56		20.44 ± 0.84			0.813		24.1 ± 0.33		24.06 ± 1.00			0.947
18:1n7		5.19 ± 0.16				5.28 ± 0.17			0.917		4.37 ± 0.16		4.15 ± 0.13			0.803		5.08 ± 0.09		5.18 ± 0.19			0.805
18:2n6		0.62 ± 0.11				0.54 ± 0.09			0.775		0.94 ± 0.11		0.92 ± 0.07			0.944		0.73 ± 0.07		0.71 ± 0.061			0.879
18:3n3		0.18 ± 0.00				0.17 ± 0.01			0.775		0.07 ± 0.00		0.07 ± 0.00			0.803		0.11 ± 0.00		0.1 ± 0.00			0.571
18:4n3		0.05 ± 0.00				0.03 ± 0.00			0.597		0.03 ± 0.00		0.03 ± 0.00			0.803		0.02 ± 0.00		0.04 ± 0.00*			0.173
20:0		0.44 ± 0.09				0.38 ± 0.03			0.775		0.23 ± 0.00		0.28 ± 0.01**			0.068		0.24 ± 0.01		0.24 ± 0.00			0.947
20:1n9		4.54 ± 0.21				4.17 ± 0.68			0.775		1.40 ± 0.11		1.41 ± 0.14			0.946		2.31 ± 0.23		2.04 ± 0.15			0.578
20:2n6		0.86 ± 0.08				0.95 ± 0.12			0.774		0.29 ± 0.03		0.26 ± 0.01			0.803		0.45 ± 0.03		0.41 ± 0.04			0.658
20:3n6		1.53 ± 0.10				1.38 ± 0.11			0.753		1.02 ± 0.07		0.9 ± 0.06			0.803		1.42 ± 0.06		1.28 ± 0.12			0.571
20:4n6		4.3 ± 0.14				4.77 ± 0.73			0.565		8.02 ± 0.15		7.82 ± 0.28			0.803		6.95 ± 0.22		7.2 ± 0.23			0.607
20:5n3		0.05 ± 0.00				0.05 ± 0.01			0.981		0.05 ± 0.01		0.03 ± 0.01			0.803		0.14 ± 0.01		0.12 ± 0.01			0.571
22:0		0.4 ± 0.12				0.31 ± 0.04			0.775		0.07 ± 0.00		0.07 ± 0.00			0.944		0.13 ± 0.01		0.11 ± 0.01			0.578
22:1n9		0.83 ± 0.10				0.73 ± 0.79			0.775		0.85 ± 0.05		0.93 ± 0.06			0.803		0.96 ± 0.16		0.8 ± 0.34			0.616
22:4n6		3.78 ± 0.17				4.00 ± 0.22			0.774		2.96 ± 0.10		2.79 ± 0.26			0.803		4.67 ± 0.08		4.55 ± 0.18			0.689
22:5n6		0.24 ± 0.02				0.22 ± 0.13			0.775		0.75 ± 0.09		0.67 ± 0.12			0.813		0.46 ± 0.02		0.38 ± 0.12			0.457
22:5n3		0.20 ± 0.01				0.27 ± 0.02*			0.546		0.3 ± 0.019		0.27 ± 0.02			0.803		0.46 ± 0.02		0.45 ± 0.03			0.814
22:6n3		1.75 ± 0.20				2 .00 ± 1.14			0.774		9.92 ± 0.33		10.55 ± 0.32			0.803		7.58 ± 0.32		7.03 ± 0.46			0.571
24:0		1.12 ± 0.11				1.04 ± 0.17			0.775		0.11 ± 0.01		0.1 ± 0.01			0.803		0.05 ± 0.01		0.19 ± 0.03**			0.049
24:1n9		3.25 ± 0.14				3.27 ± 0.45			0.996		0.51 ± 0.08		0.5 ± 0.07			0.946		1.68 ± 0.19		1.4 ± 0.20			0.571
24:5n3		0.09 ± 0.00				0.11 ± 0.02			0.597		0.06 ± 0.00		0.05 ± 0.00			0.803		0.10 ± 0.01		0.12 ± 0.01			0.457
24:6n3		0.49 ± 0.03				0.61 ± 0.07*			0.546		0.16 ± 0.02		0.13 ± 0.01			0.803		0.41 ± 0.019		0.36 ± 0.04			0.501
ACL		18.39 ± 0.02				18.43 ± 0.06			0.775		18.31 ± 0.02		18.32 ± 0.03			0.9		18.53 ± 0.00		18.46 ± 0.01**			0.049
SFA		39.27 ± 0.35				38.03 ± 0.91			0.546		46.27 ± 0.66		46.95 ± 0.42			0.803		41.15 ± 0.24		42.45 ± 0.60*			0.228
UFA		60.73 ± 0.35				61.97 ± 0.91			0.546		53.73 ± 0.66		53.05 ± 0.42			0.803		58.85 ± 0.24		57.55 ± 0.60*			0.228
MUFA		46.61 ± 0.48				46.86 ± 2.88			0.874		29.16 ± 0.89		28.57 ± 1.17			0.813		35.36 ± 0.65		34.82 ± 1.06			0.738
PUFA		14.12 ± 0.46				15.11 ± 2.03			0.597		24.57 ± 0.44		24.48 ± 0.90			0.944		23.49 ± 0.48		22.73 ± 0.60			0.571
PUFAn3		2.79 ± 0.21				3.24 ± 1.05			0.597		10.59 ± 0.33		11.12 ± 0.34			0.803		8.81 ± 0.33		8.2 ± 0.44			0.571
PUFAn6		11.33 ± 0.26				11.87 ± 0.98			0.597		13.98 ± 0.26		13.36 ± 0.61			0.803		14.68 ± 0.31		14.53 ± 0.39			0.805
DBI		103.48 ± 2.01				108.63 ± 7.70			0.574		145.19 ± 1.90		145.55 ± 3.17			0.944		142.57 ± 1.87		137.95 ± 2.42			0.457
PI		59.9 ± 2.74				65.76 ± 12.88			0.597		135.75 ± 3.00		137.92 ± 4.95			0.813		122.52 ± 3.02		117.35 ± 4.36			0.571
SFA/UFA		0.65 ± 0.00				0.61 ± 0.02			0.546		0.86 ± 0.02		0.89 ± 0.01			0.803		0.7 ± 0.00		0.74 ± 0.01*			0.228
PβOx		3.69 ± 0.52				3.29 ± 7.47			0.775		74.66 ± 13.07		92.57 ± 14.00			0.803		19.17 ± 1.47		20.76 ± 6.38			0.716
		FOREBRAIN																					
		DIENCEPHALON							SUBCORTICAL TELENCEPHALON														
		Thalamus							Hippocampus					Caudate						Putamen			
		Middle-aged		Elderly		Sig			Middle-aged		Elderly		Sig	Middle-aged		Elderly		Sig		Middle-aged		Elderly	Sig
14:0		0.67 ± 0.04		0.68 ± 0.04		0.976			0.88 ± 0.01		0.79 ± 0.18		0.759	0.63 ± 0.02		0.6 ± 0.07		0.671		0.62 ± 0.01		0.64 ± 0.05	0.809
16:0		18.08 ± 0.64		18.78 ± 0.91		0.934			20.99 ± 0.65		21.7 ± 0.65		0.759	21.46 ± 0.35		21.55 ± 0.50		0.932		19.39 ± 0.35		20.13 ± 0.57	0.568
16:1n7		1.34 ± 0.06		1.23 ± 0.14		0.761			1.26 ± 0.05		1.21 ± 0.22		0.759	0.92 ± 0.04		0.87 ± 0.05		0.673		0.89 ± 0.03		1.13 ± 0.11	0.309
18:0		23.36 ± 0.25		22.37 ± 0.59		0.687			22.01 ± 0.23		22.3 ± 0.39		0.759	25.41 ± 0.20		25.63 ± 0.98		0.693		24.05 ± 0.33		23.42 ± 0.37	0.470
18:1n9		23.06 ± 0.93		23.41 ± 1.55		0.937			20.88 ± 0.89		20.18 ± 1.21		0.759	17.43 ± 0.50		17.04 ± 0.64		0.762		18.49 ± 0.37		19.77 ± 0.98	0.470
18:1n7		4.51 ± 0.09		4.42 ± 0.30		0.934			4.69 ± 0.14		4.56 ± 0.28		0.759	4.69 ± 0.08		4.56 ± 0.11		0.682		4.48 ± 0.17		4.75 ± 0.12	0.520
18:2n6		0.66 ± 0.07		1.33 ± 0.59		0.687			0.64 ± 0.06		0.69 ± 0.05		0.759	1.03 ± 0.11		1.01 ± 0.10		0.942		1.08 ± 0.09		0.94 ± 0.21	0.632
18:3n3		0.09 ± 0.00		0.08 ± 0.00		0.761			0.06 ± 0.00		0.05 ± 0.01		0.759	0.07 ± 0.00		0.05 ± 0.00*		0.195		0.07 ± 0.00		0.07 ± 0.01	0.899
18:4n3		0.03 ± 0.00		0.04 ± 0.00		0.687			0.02 ± 0.00		0.02 ± 0.00		0.759	0.05 ± 0.00		0.04 ± 0.00		0.297		0.02 ± 0.00		0.02 ± 0.00	0.892
20:0		0.2 ± 0.00		0.33 ± 0.11		0.687			0.15 ± 0.01		0.15 ± 0.02		0.862	0.17 ± 0.00		0.2 ± 0.00*		0.195		0.17 ± 0.00		0.19 ± 0.01	0.394
20:1n9		1.74 ± 0.14		1.53 ± 0.25		0.796			1 ± 0.15		0.81 ± 0.39		0.759	0.85 ± 0.07		0.66 ± 0.10		0.297		1.03 ± 0.06		1.01 ± 0.14	0.934
20:2n6		0.36 ± 0.03		0.33 ± 0.04		0.687			0.45 ± 0.07		0.37 ± 0.08		0.759	0.17 ± 0.01		0.14 ± 0.02		0.368		0.22 ± 0.01		0.2 ± 0.02	0.763
20:3n6		0.9 ± 0.05		0.86 ± 0.08		0.796			1.25 ± 0.08		1.19 ± 0.07		0.759	1.03 ± 0.05		1.04 ± 0.07		0.935		1.03 ± 0.06		0.89 ± 0.06	0.394
20:4n6		7.76 ± 0.29		8.9 ± 1.03		0.687			8.82 ± 0.40		9.3 ± 0.70		0.759	9.23 ± 0.28		10.44 ± 0.42*		0.195		9.33 ± 0.21		9.45 ± 0.34	0.899
20:5n3		0.11 ± 0.01		0.11 ± 0.02		0.934			0.04 ± 0.01		0.04 ± 0.00		0.817	0.07 ± 0.00		0.08 ± 0.01		0.912		0.07 ± 0.01		0.06 ± 0.03	0.899
22:0		0.11 ± 0.01		0.18 ± 0.08		0.687			0.1 ± 0.01		0.09 ± 0.02		0.759	0.08 ± 0.00		0.07 ± 0.00		0.389		0.1 ± 0.00		0.09 ± 0.02	0.711
22:1n9		0.94 ± 0.03		0.81 ± 0.16		0.937			0.84 ± 0.10		0.55 ± 0.10*		0.705	1.32 ± 0.07		1.24 ± 0.12		0.671		1.02 ± 0.03		0.4 ± 0.25	0.579
22:4n6		5.12 ± 0.13		4.27 ± 0.26*		0.401			5.34 ± 0.09		4.96 ± 0.26*		0.705	4.72 ± 0.06		4.51 ± 0.11		0.297		5.38 ± 0.10		4.68 ± 0.27**	0.118
22:5n6		0.84 ± 0.08		0.6 ± 0.13		0.586			1.15 ± 0.11		1.03 ± 0.11		0.759	0.81 ± 0.06		0.63 ± 0.07		0.265		0.81 ± 0.05		0.56 ± 0.07*	0.202
22:5n3		0.34 ± 0.01		0.41 ± 0.04		0.586			0.3 ± 0.01		0.31 ± 0.02		0.759	0.28 ± 0.01		0.29 ± 0.02		0.673		0.31 ± 0.01		0.31 ± 0.01	0.987
22:6n3		7.69 ± 0.60		7.72 ± 0.96		0.937			7.64 ± 0.50		8.53 ± 0.93		0.759	8.52 ± 0.26		8.67 ± 0.34		0.839		10 ± 0.24		9.87 ± 0.57	0.899
24:0		0.34 ± 0.08		0.27 ± 0.05		0.796			0.03 ± 0.00		0.03 ± 0.09		0.759	0.17 ± 0.02		0.09 ± 0.01*		0.195		0.02 ± 0.00		0.02 ± 0.03*	0.309
24:1n9		1.39 ± 0.24		1.04 ± 0.23		0.694			1.14 ± 0.20		0.89 ± 0.20		0.759	0.69 ± 0.08		0.45 ± 0.08		0.27		1.14 ± 0.10		1.06 ± 0.20	0.899
24:5n3		0.05 ± 0.00		0.07 ± 0.00*		0.511			0.04 ± 0.00		0.04 ± 0.00		0.890	0.06 ± 0.00		0.06 ± 0.00		0.666		0.04 ± 0.00		0.06 ± 0.00	0.311
24:6n3		0.32 ± 0.04		0.25 ± 0.03		0.687			0.26 ± 0.04		0.2 ± 0.02		0.759	0.14 ± 0.01		0.09 ± 0.01*		0.195		0.26 ± 0.03		0.28 ± 0.05	0.899
ACL		18.53 ± 0.01		18.47 ± 0.01*		0.416			18.46 ± 0.01		18.44 ± 0.03		0.759	18.45 ± 0.00		18.44 ± 0.01		0.601		18.6 ± 0.01		18.51 ± 0.02**	0.167
SFA		42.75 ± 0.62		42.6 ± 1.26		0.937			44.17 ± 0.69		45.06 ± 0.71		0.759	47.91 ± 0.33		48.13 ± 0.50		0.839		44.35 ± 0.31		44.49 ± 0.91	0.913
UFA		57.25 ± 0.62		57.4 ± 1.26		0.937			55.83 ± 0.69		54.94 ± 0.71		0.759	52.09 ± 0.33		51.87 ± 0.50		0.839		55.65 ± 0.31		55.51 ± 0.91	0.913
MUFA		32.98 ± 1.34		32.44 ± 2.05		0.937			29.82 ± 1.30		28.21 ± 2.38		0.759	25.91 ± 0.71		24.82 ± 0.78		0.601		27.04 ± 0.55		28.12 ± 1.32	0.711
PUFA		24.27 ± 0.75		24.96 ± 1.06		0.796			26.01 ± 0.66		26.73 ± 1.82		0.759	26.18 ± 0.48		27.05 ± 0.47		0.535		28.61 ± 0.29		27.39 ± 0.63	0.319
PUFAn3		8.64 ± 0.58		8.68 ± 0.90		0.937			8.36 ± 0.47		9.19 ± 0.90		0.759	9.2 ± 0.24		9.28 ± 0.35		0.912		10.77 ± 0.20		10.66 ± 0.50	0.899
PUFAn6		15.64 ± 0.28		16.28 ± 1.30		0.796			17.65 ± 0.35		17.54 ± 0.94		0.863	16.98 ± 0.30		17.78 ± 0.41		0.379		17.84 ± 0.30		16.73 ± 0.29**	0.167
DBI		144.4 ± 2.97		145.16 ± 3.27		0.956			147.71 ± 2.51		150.64 ± 7.02		0.759	145.67 ± 1.56		148.21 ± 1.78		0.601		159.55 ± 0.88		155.72 ± 2.94	0.319
PI		127.6 ± 5.46		128.06 ± 6.90		0.976			133.56 ± 4.69		139.7 ± 11.29		0.759	136.66 ± 2.59		140.35 ± 2.68		0.625		152.54 ± 1.55		147.47 ± 4.76	0.470
SFA/UFA		0.75 ± 0.01		0.74 ± 0.04		0.937			0.79 ± 0.02		0.82 ± 0.02		0.759	0.92 ± 0.01		0.93 ± 0.01		0.839		0.8 ± 0.01		0.8 ± 0.03	0.899
PβOx		29.85 ± 3.86		33.91 ± 6.53		0.934			37.07 ± 6.16		48.54 ± 9.09		0.759	70.59 ± 11.74		109.16 ± 14.86		0.249		43.02 ± 5.80		48.88 ± 10.68	0.892
	FOREBRAIN																						
	CORTICAL TELENCEPHALON																						
	Occipital cortex								Parietal cortex					Temporal cortex									
	Middle-aged					Elderly	Sig		Middle-aged		Elderly		Sig	Middle-aged								Elderly	Sig
14:0	0.81 ± 0.03					0.7 ± 0.04	0.494		0.84 ± 0.04		0.7 ± 0.02*		0.557	0.82 ± 0.01								0.85 ± 0.03	0.773
16:0	21.76 ± 0.36					21.18 ± 0.37	0.825		21.11 ± 0.57		20.56 ± 0.49		0.846	24.73 ± 0.36								24.11 ± 0.83	0.503
16:1n7	1.17 ± 0.06					1.02 ± 0.13	0.705		1.3 ± 0.07		1.14 ± 0.07		0.846	0.98 ± 0.05								0.94 ± 0.10	0.773
18:0	24.52 ± 0.26					23.74 ± 0.49	0.494		24.38 ± 0.39		24.28 ± 0.38		0.973	24.87 ± 0.18								25.09 ± 0.12	0.756
18:1n9	17.89 ± 0.58					18.54 ± 0.92	0.977		19.72 ± 0.81		19.85 ± 0.63		0.973	15.11 ± 0.42								16.28 ± 0.69	0.231
18:1n7	3.94 ± 0.11					4.00 ± 0.19	0.982		4.3 ± 0.10		4.3 ± 0.26		0.996	3.33 ± 0.06								3.51 ± 0.19	0.699
18:2n6	1.05 ± 0.12					1.15 ± 0.12	0.977		1.21 ± 0.11		1.21 ± 0.13		0.996	0.94 ± 0.10								1.05 ± 0.10	0.773
18:3n3	0.06 ± 0.00					0.06 ± 0.00	0.982		0.08 ± 0.00		0.07 ± 0.00		0.846	0.04 ± 0.00								0.04 ± 0.00	0.978
18:4n3	0.03 ± 0.00					0.04 ± 0.00	0.494		0.04 ± 0.00		0.04 ± 0.00		0.973	0.03 ± 0.00								0.03 ± 0.00	0.823
20:0	0.19 ± 0.00					0.21 ± 0.00	0.494		0.21 ± 0.00		0.2 ± 0.00		0.973	0.16 ± 0.00								0.18 ± 0.00*	0.147
20:1n9	1.03 ± 0.09					1.02 ± 0.11	0.987		1.19 ± 0.16		1.04 ± 0.16		0.863	0.46 ± 0.05								0.52 ± 0.17	0.773
20:2n6	0.25 ± 0.02					0.21 ± 0.02	0.817		0.30 ± 0.03		0.25 ± 0.01		0.846	0.16 ± 0.01								0.15 ± 0.05	0.773
20:3n6	0.99 ± 0.05					0.97 ± 0.07	0.982		1.08 ± 0.054		1.02 ± 0.07		0.863	1.11 ± 0.07								1.02 ± 0.06	0.756
20:4n6	7.33 ± 0.23					7.38 ± 0.31	0.982		7.71 ± 0.32		8.02 ± 0.27		0.876	9.96 ± 0.31								9.1 ± 0.41	0.231
20:5n3	0.06 ± 0.012					0.06 ± 0.00	0.982		0.07 ± 0.00		0.06 ± 0.02		0.921	0.08 ± 0.01								0.07 ± 0.00	0.773
22:0	0.09 ± 0.00					0.10 ± 0.00	0.982		0.11 ± 0.01		0.11 ± 0.01		0.973	0.05 ± 0.00								0.06 ± 0.01	0.756
22:1n9	2.18 ± 0.10					2.28 ± 0.27	0.982		0.74 ± 0.04		0.48 ± 0.07		0.846	0.86 ± 0.39								1.07 ± 0.20	0.782
22:4n6	3.9 ± 0.13					3.51 ± 0.12	0.494		4.08 ± 0.13		3.88 ± 0.12		0.846	4.43 ± 0.11								4.04 ± 0.15*	0.173
22:5n6	0.78 ± 0.05					0.65 ± 0.09	0.592		0.84 ± 0.09		0.72 ± 0.09		0.846	0.96 ± 0.08								0.66 ± 0.06*	0.164
22:5n3	0.33 ± 0.01					0.37 ± 0.02	0.502		0.3 ± 0.01		0.34 ± 0.03		0.759	0.34 ± 0.02								0.36 ± 0.03	0.773
22:6n3	10.49 ± 0.29					11.69 ± 0.59	0.502		8.95 ± 0.43		10.37 ± 0.46		0.557	10.04 ± 0.27								10.4 ± 0.57	0.756
24:0	0.14 ± 0.019					0.13 ± 0.032	0.982		0.22 ± 0.04		0.2 ± 0.03		0.973	0.04 ± 0.00								0.06 ± 0.04	0.509
24:1n9	0.80 ± 0.10					0.80 ± 0.16	0.987		0.97 ± 0.22		0.83 ± 0.13		0.936	0.24 ± 0.06								0.28 ± 0.22	0.773
24:5n3	0.04 ± 0.00					0.03 ± 0.00	0.792		0.05 ± 0.00		0.07 ± 0.01		0.742	0.06 ± 0.00								0.04 ± 0.00*	0.147
24:6n3	0.16 ± 0.01					0.17 ± 0.02	0.982		0.19 ± 0.02		0.26 ± 0.07		0.846	0.19 ± 0.02								0.09 ± 0.03**	0.057
ACL	18.49 ± 0.01					18.54 ± 0.01*	0.494		18.42 ± 0.02		18.47 ± 0.01		0.557	18.39 ± 0.01								18.38 ± 0.02	0.773
SFA	47.51 ± 0.47					46.05 ± 0.71	0.494		46.88 ± 0.76		46.05 ± 0.78		0.846	50.68 ± 0.46								50.35 ± 0.77	0.773
UFA	52.49 ± 0.47					53.95 ± 0.71	0.494		53.12 ± 0.76		53.95 ± 0.78		0.846	49.32 ± 0.46								49.65 ± 0.77	0.773
MUFA	27.02 ± 0.85					27.66 ± 1.38	0.982		28.22 ± 1.23		27.66 ± 1.17		0.973	20.98 ± 0.71								22.6 ± 1.53	0.254
PUFA	25.48 ± 0.48					26.3 ± 0.85	0.825		24.91 ± 0.54		26.3 ± 0.43		0.646	28.34 ± 0.46								27.06 ± 0.86	0.231
PUFAn3	11.17 ± 0.29					12.43 ± 0.58	0.494		9.68 ± 0.41		11.19 ± 0.42*		0.557	10.77 ± 0.31								11.03 ± 0.57	0.773
PUFAn6	14.3 ± 0.39					13.86 ± 0.30	0.793		15.22 ± 0.36		15.11 ± 0.30		0.973	17.57 ± 0.42								16.02 ± 0.33*	0.147
DBI	147.77 ± 1.58					153.92 ± 3.38	0.500		143.21 ± 1.98		151.26 ± 1.78*		0.557	152.85 ± 1.72								149.47 ± 3.06	0.517
PI	141.57 ± 2.60					149.4 ± 5.77	0.705		132.58 ± 3.79		144.17 ± 3.66		0.557	152.02 ± 2.57								147.29 ± 5.51	0.517
SFA/UFA	0.91 ± 0.01					0.85 ± 0.024	0.494		0.89 ± 0.02		0.85 ± 0.02		0.846	1.03 ± 0.01								1.01 ± 0.02	0.773
PβOx	84.36 ± 14.11					80.57 ± 17.42	0.982		58.46 ± 12.84		60.12 ± 13.10		0.973	61.01 ± 11.706								135.92 ± 22.40**	0.057
			FOREBRAIN																				
			CORTICAL TELENCEPHALON																				
			Entorhinal cortex							Frontal cortex							Cingulate cortex						
			Middle-aged		Elderly			Sig		Middle-aged		Elderly			Sig		Middle-aged		Elderly		Sig		
14:0			0.79 ± 0.03		0.81 ± 0.03			0.866		0.84 ± 0.04		0.77 ± 0.04			0.953		0.95 ± 0.05		0.87 ± 0.03		0.934		
16:0			20.98 ± 0.66		20.03 ± 0.85			0.695		22.16 ± 0.51		22.09 ± 0.36			0.964		23.73 ± 0.93		22.52 ± 0.80		0.934		
16:1n7			1.29 ± 0.07		1.52 ± 0.17			0.691		1.38 ± 0.08		1.28 ± 0.08			0.953		1.38 ± 0.12		1.52 ± 0.15		0.934		
18:0			24.38 ± 0.27		23.82 ± 0.36			0.691		24.33 ± 0.26		24.28 ± 0.49			0.964		22.79 ± 0.41		22.49 ± 0.42		0.934		
18:1n9			18.74 ± 0.95		20.86 ± 1.28			0.691		17.94 ± 0.84		18.03 ± 0.56			0.964		18.56 ± 1.18		19.53 ± 0.87		0.934		
18:1n7			4.05 ± 0.16		4.11 ± 0.22			0.908		3.91 ± 0.11		3.85 ± 0.25			0.964		3.97 ± 0.11		4.14 ± 0.28		0.934		
18:2n6			0.78 ± 0.08		0.83 ± 0.09			0.866		1.13 ± 0.07		1.12 ± 0.10			0.964		0.81 ± 0.10		0.86 ± 0.08		0.934		
18:3n3			0.05 ± 0.00		0.06 ± 0.00			0.695		0.06 ± 0.00		0.05 ± 0.00			0.953		0.06 ± 0.00		0.07 ± 0.00		0.934		
18:4n3			0.03 ± 0.00		0.03 ± 0.00			0.759		0.04 ± 0.00		0.03 ± 0.00			0.953		0.03 ± 0.00		0.03 ± 0.00		0.934		
20:0			0.21 ± 0.00		0.23 ± 0.01			0.691		0.25 ± 0.08		0.19 ± 0.01			0.953		0.19 ± 0.00		0.22 ± 0.00**		0.376		
20:1n9			0.88 ± 0.15		1.06 ± 0.14			0.705		0.82 ± 0.10		0.76 ± 0.09			0.964		0.94 ± 0.23		1.07 ± 0.18		0.934		
20:2n6			0.31 ± 0.04		0.32 ± 0.03			0.975		0.24 ± 0.02		0.21 ± 0.03			0.953		0.3 ± 0.04		0.28 ± 0.04		0.934		
20:3n6			1.02 ± 0.07		0.97 ± 0.06			0.851		1.05 ± 0.04		0.99 ± 0.10			0.953		1 ± 0.05		0.98 ± 0.07		0.970		
20:4n6			8.91 ± 0.37		8.17 ± 0.34			0.691		8.38 ± 0.30		8.56 ± 0.28			0.964		8.12 ± 0.30		7.75 ± 0.35		0.934		
20:5n3			0.06 ± 0.00		0.06 ± 0.01			0.866		0.08 ± 0.01		0.05 ± 0.00			0.953		0.06 ± 0.01		0.05 ± 0.01		0.934		
22:0			0.09 ± 0.01		0.12 ± 0.01			0.691		0.08 ± 0.00		0.08 ± 0.00			0.953		0.08 ± 0.01		0.09 ± 0.01		0.934		
22:1n9			0.7 ± 0.04		0.62 ± 0.06			0.691		1.04 ± 0.12		0.96 ± 0.07			0.964		0.75 ± 0.07		0.72 ± 0.07		0.934		
22:4n6			4.94 ± 0.10		4.75 ± 0.17			0.691		4.1 ± 0.11		3.91 ± 0.23			0.953		4.72 ± 0.14		4.48 ± 0.15		0.934		
22:5n6			0.88 ± 0.05		0.68 ± 0.13			0.691		0.96 ± 0.10		0.73 ± 0.16			0.953		1.03 ± 0.12		0.78 ± 0.08		0.934		
22:5n3			0.3 ± 0.01		0.31 ± 0.02			0.908		0.31 ± 0.01		0.34 ± 0.02			0.953		0.3 ± 0.02		0.32 ± 0.02		0.934		
22:6n3			9.29 ± 0.44		8.72 ± 0.78			0.763		9.97 ± 0.49		10.87 ± 0.53			0.953		9.08 ± 0.62		9.84 ± 0.57		0.934		
24:0			0.23 ± 0.053		0.34 ± 0.07			0.691		0.16 ± 0.03		0.14 ± 0.02			0.964		0.2 ± 0.08		0.22 ± 0.04		0.970		
24:1n9			0.85 ± 0.19		1.32 ± 0.31			0.691		0.58 ± 0.17		0.54 ± 0.11			0.964		0.74 ± 0.29		0.93 ± 0.24		0.934		
24:5n3			0.04 ± 0.00		0.05 ± 0.00			0.695		0.05 ± 0.00		0.04 ± 0.00			0.953		0.04 ± 0.00		0.04 ± 0.00		0.934		
24:6n3			0.17 ± 0.03		0.23 ± 0.04			0.695		0.13 ± 0.02		0.12 ± 0.01			0.964		0.15 ± 0.04		0.18 ± 0.03		0.934		
ACL			18.48 ± 0.01		18.48 ± 0.01			0.975		18.43 ± 0.00		18.44 ± 0.01			0.953		18.38 ± 0.02		18.42 ± 0.01		0.934		
SFA			46.68 ± 0.82		45.36 ± 1.07			0.691		47.83 ± 0.60		47.56 ± 0.52			0.964		47.95 ± 1.10		46.41 ± 0.91		0.934		
UFA			53.32 ± 0.82		54.64 ± 1.07			0.691		52.17 ± 0.60		52.44 ± 0.52			0.964		52.05 ± 1.10		53.59 ± 0.91		0.934		
MUFA			26.52 ± 1.39		29.48 ± 2.00			0.691		25.68 ± 1.20		25.42 ± 0.86			0.964		26.34 ± 1.90		27.93 ± 1.58		0.934		
PUFA			26.8 ± 0.58		25.16 ± 0.95			0.691		26.49 ± 0.68		27.02 ± 0.41			0.953		25.71 ± 0.85		25.67 ± 0.69		0.970		
PUFAn3			9.95 ± 0.41		9.45 ± 0.73			0.787		10.63 ± 0.47		11.5 ± 0.53			0.953		9.73 ± 0.59		10.53 ± 0.56		0.934		
PUFAn6			16.85 ± 0.26		15.71 ± 0.33*			0.691		15.86 ± 0.32		15.52 ± 0.63			0.953		15.98 ± 0.34		15.13 ± 0.46		0.934		
DBI			150.66 ± 2.00		145.79 ± 3.38			0.691		149.4 ± 2.67		152.96 ± 1.19			0.953		145.79 ± 2.83		148.5 ± 2.14		0.934		
PI			142.85 ± 4.21		133.85 ± 6.83			0.691		143.49 ± 4.71		148.97 ± 2.63			0.953		137.84 ± 5.74		140.32 ± 4.55		0.934		
SFA/UFA			0.88 ± 0.02		0.83 ± 0.03			0.691		0.92 ± 0.02		0.91 ± 0.01			0.964		0.93 ± 0.03		0.87 ± 0.03		0.934		
PβOx			79.71 ± 15.08		58.42 ± 20.69			0.732		91.97 ± 13.39		105.31 ± 16.42			0.953		84.06 ± 12.88		75.12 ± 18.50		0.934		

### Brain regional changes in fatty acid profile: comparison of middle-aged and elderly groups

Comparison of middle-aged and elderly groups throughout the different brain regions analyzed verified the existence of minor changes (Table 2). Thus, in hindbrain, *olive* showed a decrease in 14:0 (30%, *p* < 0.05) and increase in 22:5n-3 (30%, *p* < 0.05) and 24:6n-3 (25%, *p* < 0.05), fatty acids with a very low abundance, and no involvement in general indexes in the elderly group compared to the middle-aged group, whilst *vermis* only showed an increase in 20:0 (22%, *p* < 0.01) in the elderly group, without any additional change. In the midbrain, and in a similar way to hindbrain, *substantia nigra* only showed an increase in 16:1n-7 (10%, *p* < 0.05), and the very minor fatty acids 18:4n-3 (100%, *p* < 0.05) and 24:0 (280%, *p* < 0.01), as well as SFA content (3%, *p* < 0.05), and a decrease in ACL (0.5%, *p* < 0.01) and UFA (3%, *p* < 0.05).

In diencephalon, *thalamus* showed a decrease in 22:4n-6 (17%, *p* < 0.05) content and ACL (0.5%, *p* < 0.05), and an increase in 24:5n-3 (40%, *p* < 0.05) content in the elderly group compared to the middle-aged group. In subcortical telencephalon, the three analyzed regions, hippocampus, caudate, and putamen, also showed minor changes in the elderly group. Thus, in *hippocampus*, we only found a decrease in the relative abundance of fatty acids 22:1n-9 (35%, *p* < 0.05) and 22:4n-6 (7%, *p* < 0.05); in *caudate nucleus* a decrease in 18:3n-3 (30%, *p* < 0.05), 24:0 (47%, *p* < 0.05), and 24:6n-3 (36%, *p* < 0.05), and an increase in the content of 20:0 (17%, *p* < 0.01) and 20:4n-6 (13%, *p* < 0.05); and in the *putamen nucleus*, a decrease in 22:4n-6 (13%, *p* < 0.01), 22:5n-6 (30%, *p* < 0.05), and PUFAn-6 (6%, *p* < 0.01) contents, as well as ACL (0.5%, *p* < 0.01).

Finally, six regions from human cerebral cortex (cortical telencephalon) were analyzed, again showing minor changes in the elderly group. Thus, in *occipital cortex* (areas 17–18), fatty acid composition analysis revealed a slight increase in the ACL (0.3%, *p* < 0.05) in the elderly group compared to the middle-aged group; in *parietal cortex* (area 7), a decrease in 14:0 (17%, *p* < 0.05) content, and increase in PUFAn-3 (15%, *p* < 0.05) and DBI (6%, *p* < 0.05) was observed; in *temporal cortex* (inferior temporal area 20), a decrease in 22:4n-6 (9%, *p* < 0.05), 22:5n-6 (31%, *p* < 0.05), 24:5n-3 (33%, *p* < 0.05), 24:6n-3 (53%, *p* < 0.01), and PUFAn-6 (9%, *p* < 0.05), and an increase in 20:0 (12%, *p* < 0.05) and estimation of the peroxisomal β-oxidation activity (123%, *p* < 0.001) was detected; in *entorhinal cortex*, the only observed change was a decrease in PUFAn-6 content (7%, *p* < 0.05); in *frontal cortex* (area 8), no change was verified; and finally, in *cingulate cortex* (area 24), only a slight increase in 20:0 (15%, *p* < 0.01) was revealed.

Because of the large number of tests, adjustment for multiple comparisons was assessed using the Benjamini–Hochberg false discovery rate (FDR) analysis. *p* values when comparing the two groups of cases is shown in Table 2. As a result, only 24:0 and ACL in substantia nigra, and 24:6n-3 and peroxisomal β-oxidation in the temporal cortex were sustained following multiple comparison adjustment.

### Brain regional changes in estimated desaturase and elongase activity: general traits

Since some of the observed changes in the elderly group may be due to alterations in the activity of the enzymes involved in the fatty acid biosynthesis, elongase and desaturase activity was estimated from specific product/substrate ratios. The outcomes confirm the existence of minor changes induced by aging across the different brain regions (Supplementary Table S1). Overall, the higher estimated desaturase activity corresponds first to delta-5-desaturase (D5D, r20:4n-6/20:3n-6), and then to delta-6-desaturase (b) (D6D (b), r24:6n-3/24:5n-3), with D5D being systematically higher than D6D in all brain regions with the only exception of olive (medulla oblongata), where D6D is higher than D5D. For elongases, the higher activity corresponds to Elovl2-5 (n-3) (r22:5n-3/20:5n-3) followed by Elovl3 (n-9) (c) (r24:1n-9/22:1n-9) and Elovl6 (r18:0/16:0); this observation is maintained in all brain regions.

### Brain regional changes in estimated desaturase and elongase activity: comparison of middle-aged and elderly groups

The changes observed on the elderly compared with the middle-aged group were region-dependent. Specifically, in the hindbrain, a reduced Elovl1-3–7 (a) (*p* < 0.01) and (b) (*p* < 0.05) estimated activity in *vermis* was observed. In the midbrain, *substantia nigra* showed an increased estimated D6D (a) (*p* < 0.05), Elovl1-3–7 (c) (*p* < 0.001), and Elovl2 activity (*p* < 0.05), and decrease in D6D (b) activity (*p* < 0.05). In the diencephalon, *thalamus* D6D (b) and Elovl2-5 (n-6) estimated activity was decreased (*p* < 0.05 and *p* < 0.05, respectively). In the subcortical telencephalon, a reduced estimated activity of Elovl1-3–7 (b) and (c) (*p* < 0.01 and *p* < 0.05, respectively), Elovl3 (a) (*p* < 0.05), and Elovl2-5(n-6) (p < 0.05), and very slight increase of Elovl1-3–7 (a) (p < 0.05) activity were found in the *caudate nucleus*, whereas in the *putamen nucleus* Elovl1-3–7 (b) activity was decreased (*p* < 0.05) along with an enhanced Elovl1-3–7 (c) and Elovl2 activity (both *p* < 0.05). Finally, in the six regions of the human cerebral cortex (cortical telencephalon), little changes were observed and included a very small increase in Elovl1-3–7 (a) activity (*p* < 0.05) in the *occipital cortex*, reduced D6D (b) (*p* < 0.05) and Elovl2 (*p* < 0.05) along with an enhanced Elovl6 (*p* < 0.05) and Elovl1-3–7 (a) (*p* < 0.05) estimated activity in the *temporal cortex*, and a slight increase of Elovl1-3–7 (a) (*p* < 0.05) in *cingulate cortex*. No changes in the estimated enzymatic activity were detected in *olive* (hindbrain), *hippocampus* (*subcortical telencephalon*), and *parietal*, *entorhinal*, and *frontal cortex* (cortical telencephalon). These results suggest a global increase of the Elovl1-3–7 (a) along with a reduced Elovl1-3–7 (b) and D6D (b) estimated activities in the elderly.

Because of the large number of tests, adjustment for multiple comparisons was assessed using the Benjamini–Hochberg false discovery rate (FDR) analysis. *p* values when comparing the two groups of cases are shown in Table S1. As a result, only elovl3-7 (b) and (c) and elovl2 in putamen were sustained following multiple comparison adjustment.

### Brain regional changes in lipid composition as a function of age

**Fig. 1:**
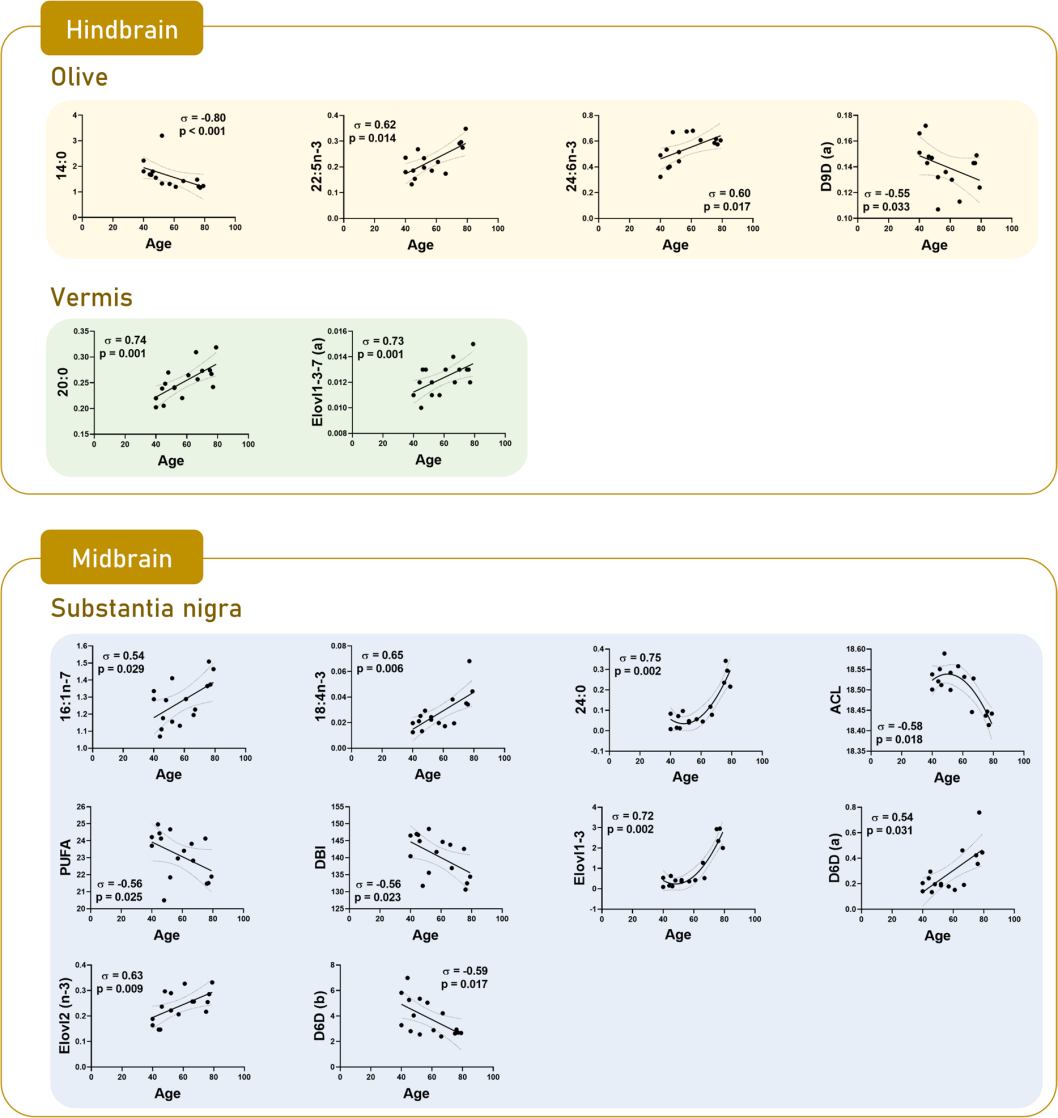
Scatterplots by region based on significant correlations between fatty acids and calculated indexes and age in hindbrain and midbrain: olive, vermis, and substantia nigra

**Fig. 2:**
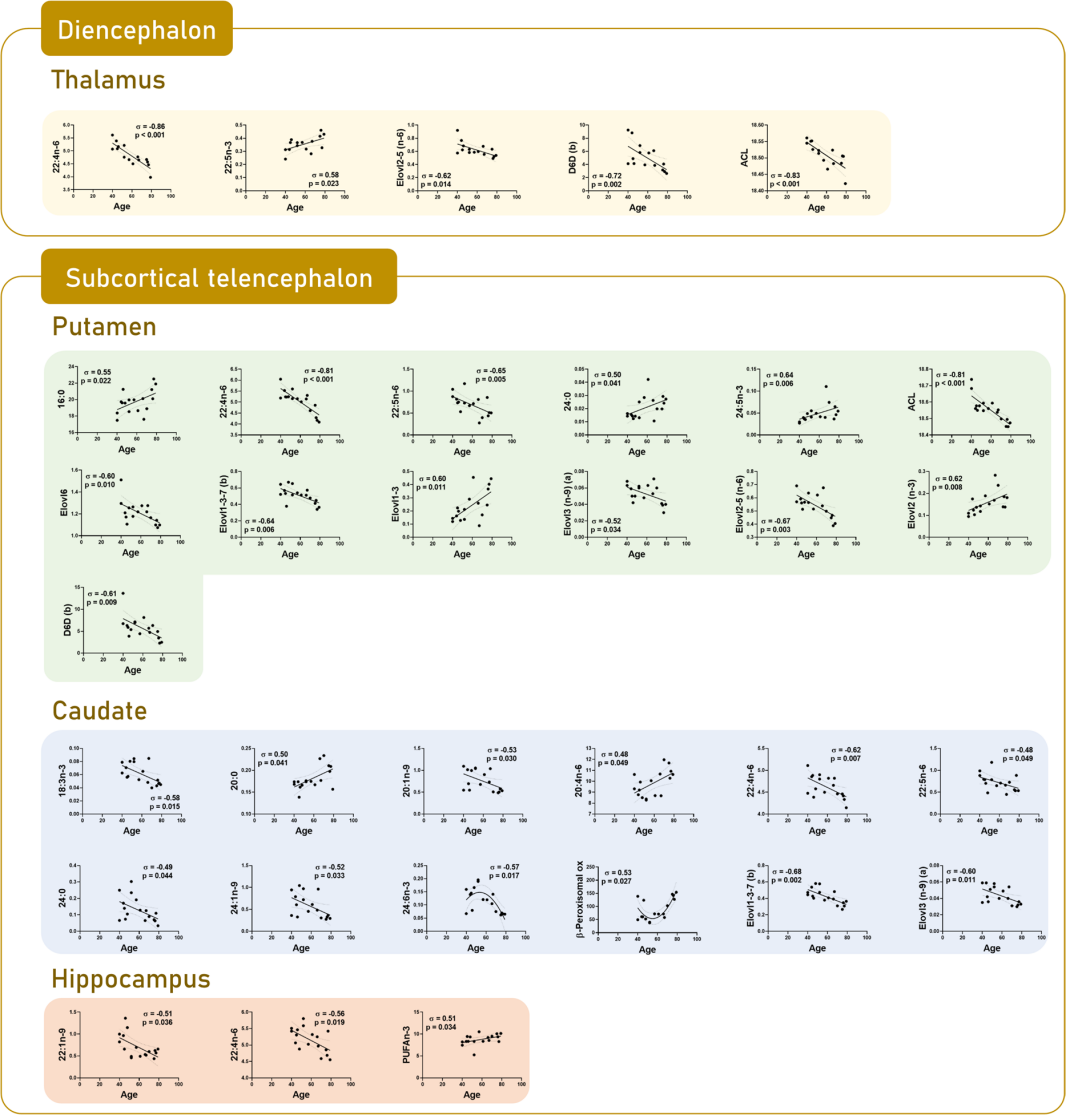
Scatterplots by region based on significant correlations between fatty acids and calculated indexes and age in diencephalon and subcortical telencephalon: thalamus, anterior putamen, head of the caudate, and hippocampus

**Fig. 3:**
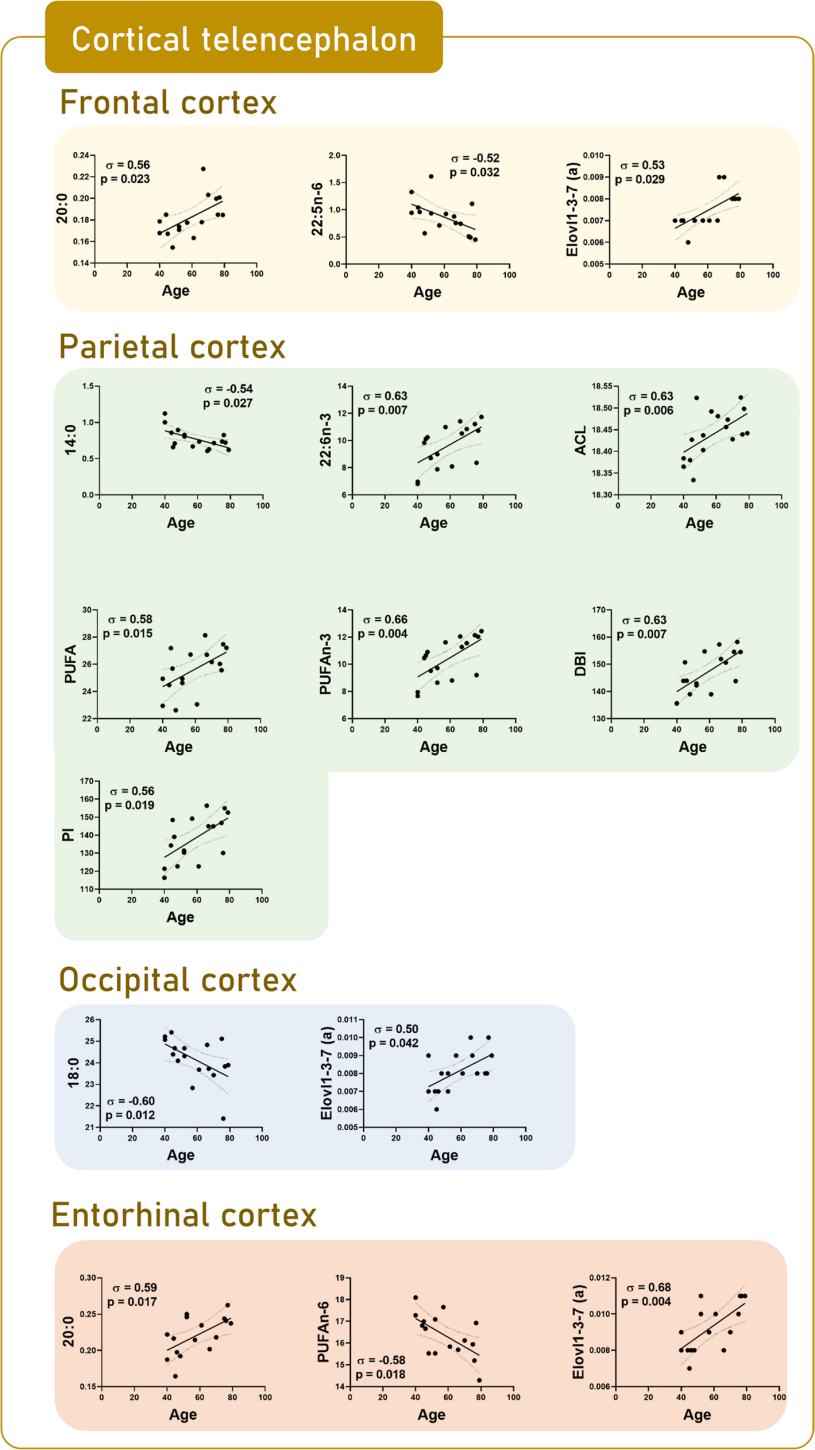
Scatterplots by region based on significant correlations between fatty acids and calculated indexes and age in cortical telencephalon: frontal cortex area 8, parietal cortex area 7, occipital cortex areas 17–18, and entorhinal cortex

**Fig. 4:**
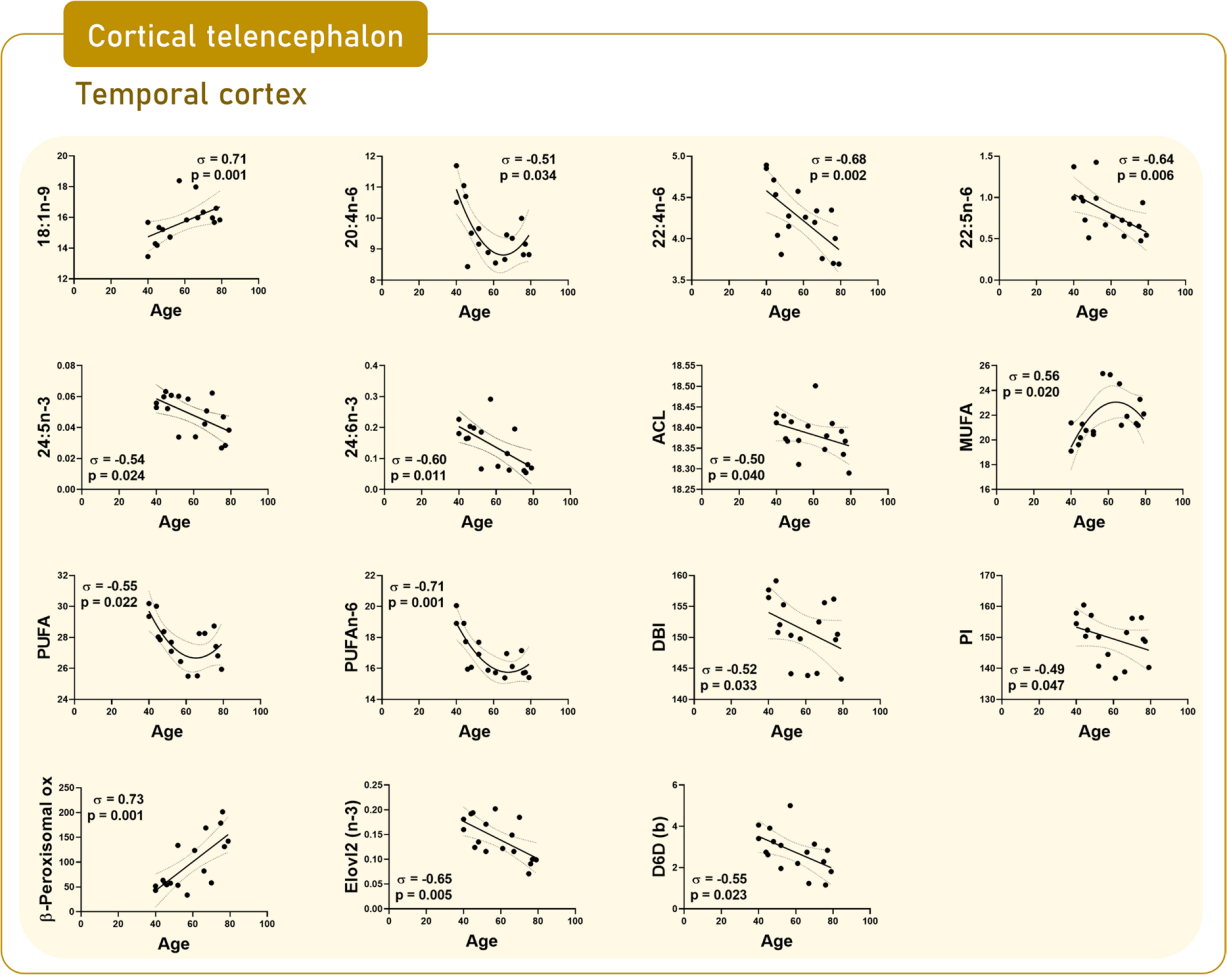
Scatterplots by region based on significant correlations between fatty acids and calculated indexes and age in cingulate gyrus (area 24)

**Fig. 5:**
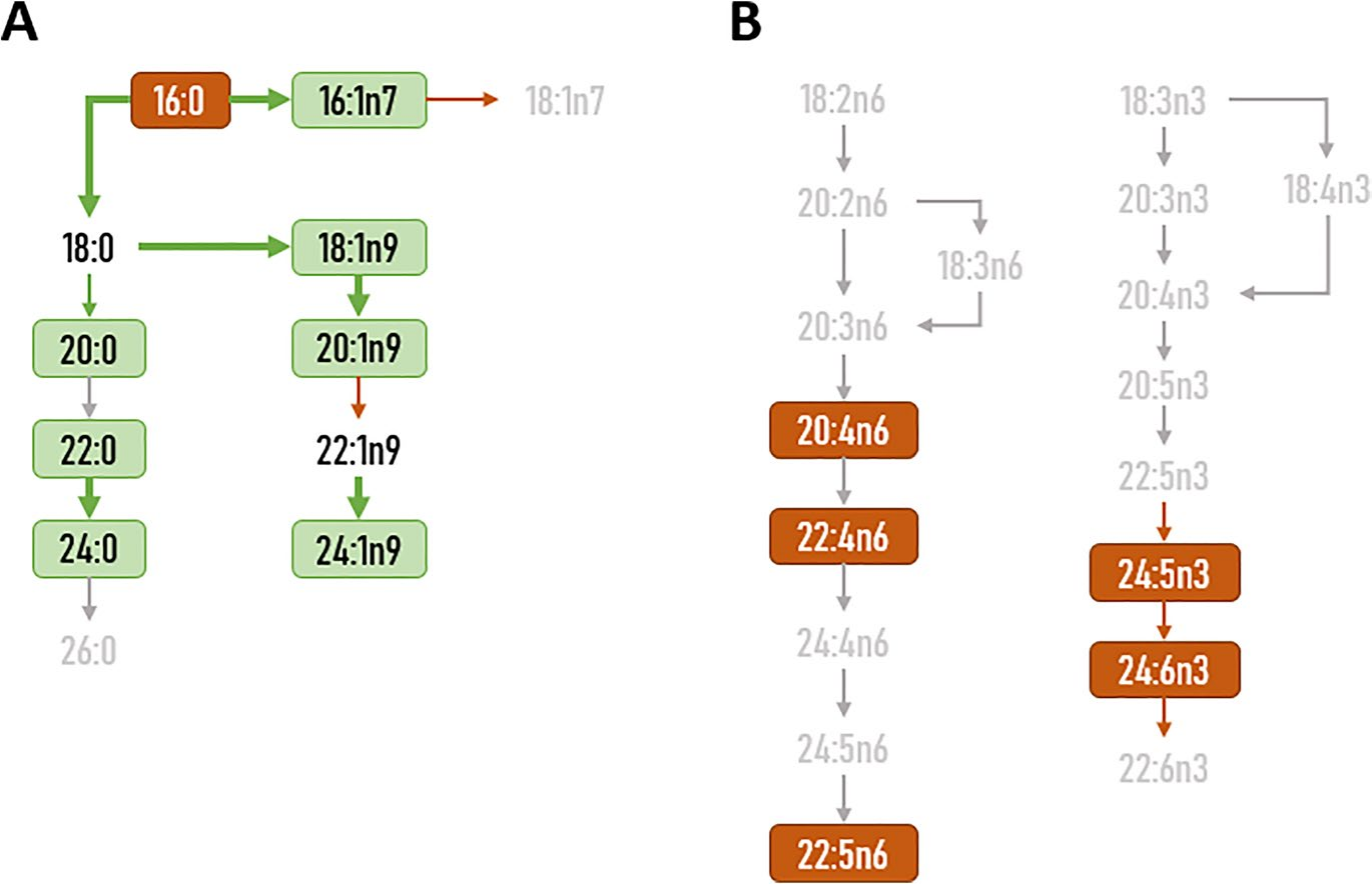
Cingulate cortex (**A**) and temporal cortex (**B**) changes in fatty acid composition associated with the aging process. Figure is based on data presented in Tables 1 and 2 and Figs. 4 and 6. Green boxes and rows represent increased content of individual FA or enhanced estimated activity in aged individuals compared to the middle-aged. Orange boxes and rows represent reduced content of individual FA or decreased estimated activity in aged individuals compared to the middle-aged. Gray text and rows represent unchanged content of individual FA or estimated activity in aged individuals compared to the middle-aged

**Fig. 6:**
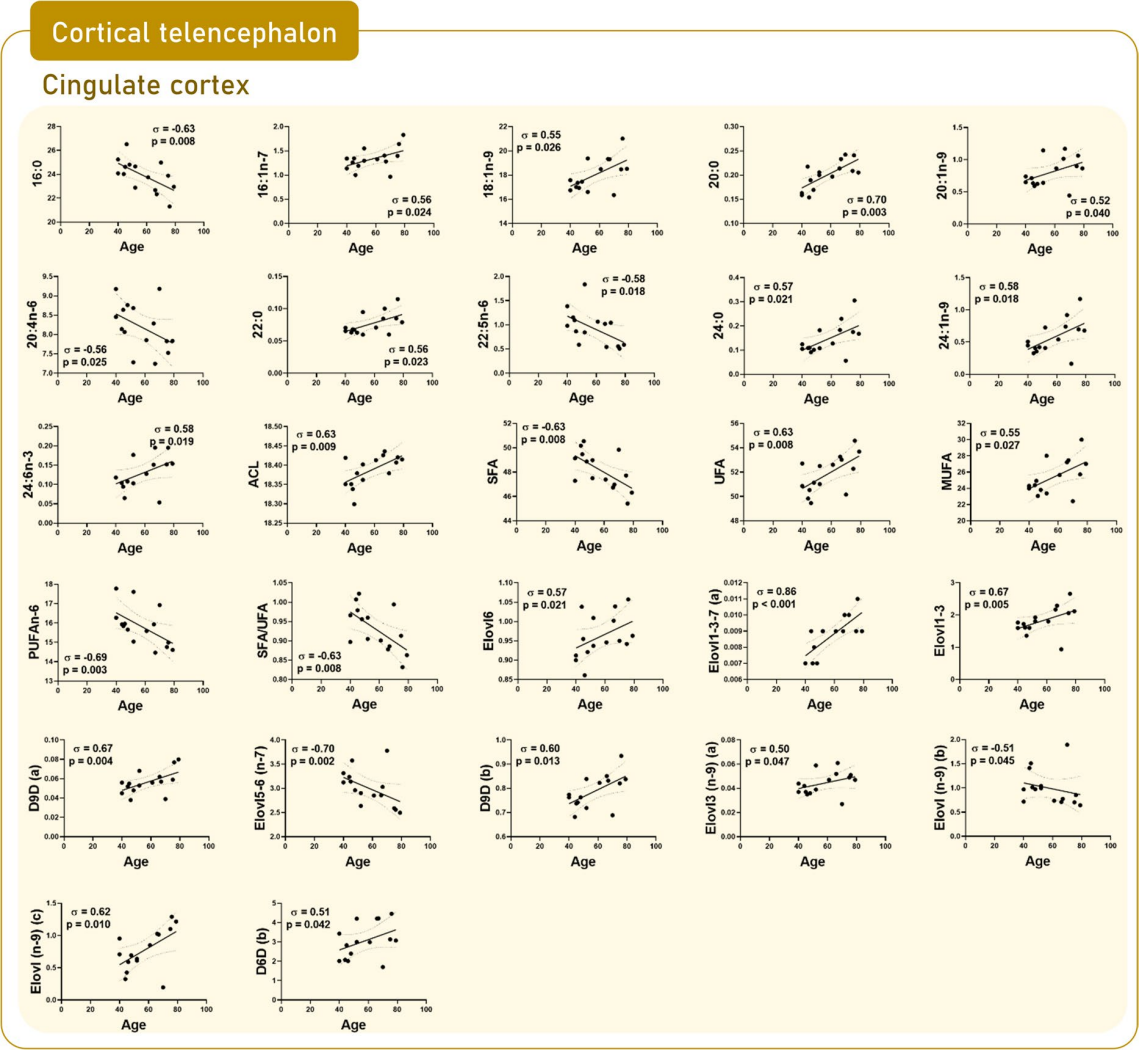
Scatterplots by region based on significant correlations between fatty acids and calculated indexes and age in inferior temporal cortex (area 20)

**Fig. 7:**
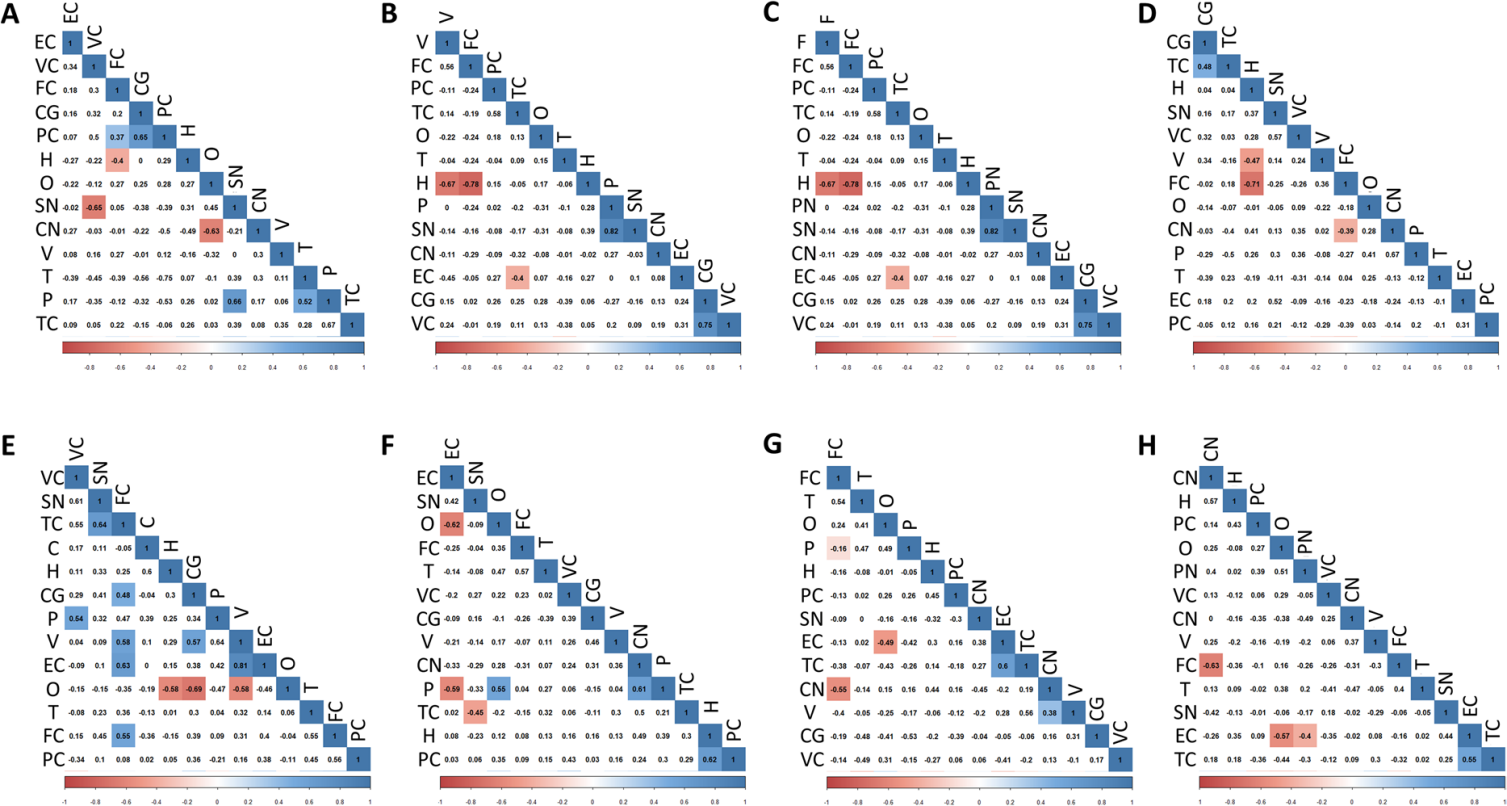
Correlation matrix for integrative indexes calculated from fatty acid composition to evaluate the existence of regional relationships. **A** Average chain length (ACL); **B** saturated fatty acids (SFA); **C** Unsaturated fatty acids (UFA); **D** monounsaturated fatty acids (MUFA); **E** polyunsaturated fatty acids n-6 series (PUFAn6), **F** polyunsaturated fatty acids series n-3 (PUFAn3); **G** double bond index (DBI); **H** peroxidizability index (PI). Brain regions: CG, cingulate gyrus; CN, caudate nucleus; EC, entorhinal cortex; FC, frontal cortex; H, hippocampus; O, olive; P, putamen; PC, parietal cortex; SN, substantia nigra; T, thalamus; TC, temporal cortex; V, vermis; VC, visual (occipital) cortex

An additional approach was used to learn about the lipid composition changes as a function of age, as a continuous variable instead of dichotomizing the data into a middle-aged and an elderly cohort. To this end, the existence of correlations between fatty acid composition and age was evaluated by applying a Spearman correlation test. Results are presented in Supplementary Table S2 and Figs. 1, 2, 3, 4, 5, 6, and 7. Remarkably, in addition to previously observed changes in fatty acid composition with age comparing middle-aged vs elderly groups (also confirmed by the correlation analysis), new relationships appeared as a continuum with the correlation analysis in diverse brain regions. Reinforcing our findings, potential interference of the variables of gender and post-mortem time (PMT) in the age-based trajectories of the different fatty acids and indexes was ruled out after applying the corresponding statistical analysis (data not shown).

More specifically, in the hindbrain, the correlation analysis did not provide additional changes with age in *olive* and *vermis* (Fig. 1). In the midbrain, in *substantia nigra*, an additional negative correlation was detected in PUFA (*p* = 0.025) content and DBI (*p* = 0.023) with age (Fig. 1). In the diencephalon, in *thalamus*, an increase in the content of 22:5n-3 (*p* = 0.023) was added (Fig. 2). In the subcortical telencephalon, in the *hippocampus*, an increase in PUFAn-3 (*p* = 0.034), and in the *putamen*, an increase with age in the content of 16: 0 (*p* = 0.022) and 24:5n-3 (*p* = 0.006) was additionally observed (Fig. 2). Especially noteworthy is the amplitude of the additional change detected in the *caudate*, with a decrease in the content of the fatty acids 20:1n-9 (*p* = 0.03), 22:4n-6 (*p* = 0.007), 22:5n-6 (*p* = 0.049), and 24:1n-9 (*p* = 0.033) (Fig. 2). At the cortical telencephalon level, the following new correlations should be highlighted: in *occipital cortex*, decrease in content of 18:0 (*p* = 0.012); in *parietal cortex*, decrease in content of 14:0 (*p* = 0.027), and increase in content of 22:6n-3 (*p* = 0.007) and, concomitantly, ACL (*p* = 0.006), PUFA (*p* = 0.015), and PI (*p* = 0.019); in *entorhinal cortex*, the increase in 20:0 (*p* = 0.017); and in *frontal cortex*, the increase in 20:0 (*p* = 0.023), and the decrease in 22:5n-6 (*p* = 0.018) (Fig. 3). *Cingulate cortex* and *temporal cortex* require special mention.

*Cingulate cortex*, which is located in the medial region of the cortical telencephalon, appears to be the region most affected by the aging process out of the thirteen included in the present study (Tables 2, S1 and S2, and Fig. 4). Thus, in addition to the increased content of 20:0 (*p* < 0.01) detected in the comparison between middle-age vs elderly individuals, the following new correlations should be added: decreased content with age of 16:0 (*p* = 0.008), 20:4n-6 (*p* = 0.025), 22:5n-6 (*p* = 0.018), SFA (*p* = 0.008), PUFAn-6 (*p* = 0.003), and ratio SFA/UFA (*p* = 0.008); and increased content with age of 16:1n-7 (*p* = 0.024), 18:1n-9 (*p* = 0.026), 20:1n-9 (*p* = 0.04), 22:0 (*p* = 0.023), 24:0 (*p* = 0.021), 24:1n-9 (*p* = 0.018), 24:6n-3 (*p* = 0.019), ACL (*p* = 0.009), UFA (*p* = 0.008), and MUFA (*p* = 0.027). Globally, these changes may be attributed to enhanced metabolic activity of the elongases and desaturases involved in the biosynthesis of SFA and MUFA from 16:0 (Table S2 and Fig. 5A).

Fatty acid composition of an additional region located in the cortical telencephalon, the *inferior temporal cortex*, is also profoundly affected by the aging process (Tables 2, S1 and S2, and Fig. 6). Thus, aside from the changes detected in the comparison between middle-age and elderly individuals, the correlation analysis did provide additional changes with age. Thus, a decrease in the content of 20:4n-6 (*p* = 0.034), 22:4n-6 (*p* = 0.002), 22:5n-6 (*p* = 0.006), 24:5n-3 (*p* = 0.024), 24:6n-3 (*p* = 0.011), ACL (*p* = 0.04), PUFA (*p* = 0.022), PUFAn-6 (*p* = 0.001), DBI (*p* = 0.033), and PI (*p* = 0.047) was observed with age; while 18:1n-9 (*p* = 0.001), MUFA (*p* = 0.02), and peroxisomal β-oxidation activity (*p* = 0.001) showed an increase with age. All these changes are suggestive of alterations with age in the PUFAn-6 biosynthesis pathway, as well as at the peroxisomal level (Fig. 5B).

Adjustment for multiple correlations was assessed using the Benjamini–Hochberg false discovery rate (FDR) analysis. *p* values at FDR 10% are shown in Table 3 and Table S2. After correction, fatty acid content and derived indexes in *substantia nigra*, *hippocampus*, *caudate*, *occipital cortex*, *parietal cortex*, *entorhinal cortex*, *frontal cortex*, and age as a continuum were no longer significant. Significant correlations were maintained in *olive* for 14:0 (*p* = 0.001); in *vermis* for 20:0 (*p* = 0.035); in *thalamus* for 22:4n-6 (*p* = 0.001) and ACL (*p* = 0.001); in *putamen* for 22:4n-6 (*p* = 0.001) and ACL (*p* = 0.001); in *temporal inferior cortex* for 18:1n-9 (*p* = 0.018), 22:4n-6 (*p* = 0.023) and PUFAn-6 (*p* = 0.018), and in *cingulate cortex* for 16:0 (*p* = 0.045), 20:0 (*p* = 0.045), ACL (*p* = 0.045), SFA (*p* = 0.045), UFA (*p* = 0.045), PUFAn-6 (*p* = 0.045), and ratio SFA/UFA (*p* = 0.045).

**Table 3:** Statistics of the FDR adjustment for multiple correlations

	Brain region	Variable	rho	*P* value (original)	*P* value at FDR 10%
Hindbrain	*Olive*	14:0	− 0.801	0.001	0.001
	*Vermis*	20:0	0.745	0.001	0.035
Diencephalon	*Thalamus*	22:4n6	− 0.862	0.001	0.001
		ACL	− 0.823	0.001	0.001
Subcortical telencephalon	*Putamen*	22:4n6	− 0.810	0.001	0.001
		ACL	− 0.812	0.001	0.001
Cortical telencephalon	*Temporal cortex*	18:1n9	0.709	0.001	0.018
		22:4n6	− 0.685	0.006	0.023
		PUFAn6	− 0.708	0.001	0.018
	*Cingulate cortex*	16:0	− 0.630	0.008	0.045
		20:0	0.700	0.003	0.045
		ACL	0.630	0.009	0.045
		SFA	− 0.640	0.008	0.045
		UFA	0.640	0.008	0.045
		PUFAn6	− 0.690	0.003	0.045
		SFA/UFA	− 0.640	0.008	0.045

*p* value threshold at FDR 10%. *ACL*, average chain length; *SFA*, saturated fatty acids; *UFA*, unsaturated fatty acids; *PUFAn6*, polyunsaturated fatty acids series n6. Only significant differences are indicated. For more information, see Table S2

Finally, we have developed a series of correlation matrixes for integrative indexes in order to evaluate the existence of regional relationships (Fig. 7). The results show that the regional relationships are very restricted without apparent general patterns. The index with the greatest regional relationships is PUFAn-6.

## Discussion

The human brain aging process induces changes at all levels of biological organization, although with a heterogeneous interregional impact, which demands adaptive responses in order to preserve the composition and function within physiological limits. The lipid bilayer that composes neuronal and glial cell membranes is not on the sidelines; consequently, the longer the optimal membrane lipid profile is sustained, the better neural cell function and survival.

The first evidence of changes in the lipid profile in the human brain during aging revealed, analyzing the whole brain, that there is a slow and progressive decrease with age in the total lipid content from the second-third decade of life [17–19]. Later on, different studies analyzing specific lipid classes (mostly glycerophospholipids) in diverse regions of human brain confirmed the occurrence of age-related lipid changes in terms of a decreased phospholipid content which is again very slow and progressive throughout the adult lifespan and varying in a region-dependent way [20, 21], but is accelerated at advanced age (over 80 years old) [21–24]. Furthermore, human brain cholesterol content also showed a very similar behavior with age [21, 24]. More recent studies analyzing the microsomal and mitochondrial lipidome, particularly the main phospholipid classes, of entorhinal cortex, frontal cortex, and hippocampus of subjects from 20 to 100 years old, found that minor fractions of phospholipids specifically containing PUFAn-6 slightly decrease during adult life, while phospholipid species containing PUFAn-3 increase during the same period [25–27]. In this line of minor changes with age, and even compositional stability during adult life, no significant changes were observed in the different classes of lipids and composition in fatty acids of human frontal cortex membrane microdomains (lipid rafts) in subjects with an age range between 24 and 85 years [28].

The fatty acid profile and its changes with aging have also been the subject of study in various works. In these studies, different regions of the human cerebral cortex such as frontal cortex (area 8) [29, 30], prefrontal cortex [26], orbitofrontal cortex (area 10) [31], entorhinal cortex [27], and hippocampus [25] of healthy adults with an age ranging from 20 to 80 years were analyzed. Globally, the outcomes of these studies suggest a general preservation of the fatty acid composition during adult life with minor changes, if at all, preferentially expressed as a decrease in PUFAn-6 content, and maintenance or slight increase in PUFAn-3 content, with and eventual fall at more advanced ages. Our findings reinforce these previous observations in frontal cortex, entorhinal cortex, and hippocampus, and extend this relative preservation of the fatty acid profile to other human brain regions such as olive (medulla oblongata), upper vermis (cerebellum), substantia nigra, thalamus, head of the caudate nucleus, anterior putamen, occipital (visual) cortex, and parietal cortex. In contrast, cingulate gyrus and inferior temporal cortex showed an entirely different tendency, with broad involvement of their fatty acid profiles.

Inferior temporal cortex area 20 and cingulate gyrus area 24 are the two brain regions most affected by aging, showing the most extensive changes in fatty acid profiles. Notably, the two regions share some changes, while others are region specific, highlighting the increased content in MUFAs, ascribed to the 18:1n-9 acyl chain, and decreased content of PUFAn-6, basically due to a decreased content of 20:4n-6 and 22:5n-6. While the 18:1n-9 increase could be attributed to an increase in D9D activity, the 20:4n-6 and 22:5n-6 decreases do not appear to depend on changes in the desaturase and elongase activity of their biosynthesis pathway, so the alteration in their content should be attributed to potential increases in its consumption [32], since they are fatty acids that act as substrates for the biosynthesis of eicosanoids and resolvins, respectively [9]. The consequences of these specific fatty acid profile changes are multiple. Thus, the changes in MUFAs and PUFAn-6 abundance can have repercussions at two levels: the first of them with implications in the geometric properties of lipids that can negatively affect functions such as exocytosis and formation of membrane microdomains [4]; and as to the second, the roles of affected PUFAn-6 are key in the generation of lipid mediators and, in particular, the synthesis of bioactive lipids with anti-inflammatory and neuroprotective properties that ensure cell survival and normal functioning during normal aging [9, 33]. Therefore, we hypothesize that the changes at the molecular and cellular levels described during human aging in the inferior frontal cortex [34, 35] and cingulate cortex [36]—which can give rise to functional losses in high-level visual processing and recognition memory, as well as emotional regulation, attention, and the integration of emotional and cognitive processes, respectively—may have as a substrate, at least in part, the alterations of the lipid profile described in this study. However, the underlying molecular mechanisms that determine the high susceptibility of these brain areas to the aging process instead of other regions is a question that remains unknown and more studies are needed to clarify this finding.

In contrast to the compositional sustainability with aging that characterizes most brain areas, in pathological conditions such as Alzheimer’s disease (AD) a marked change in temporal trajectory of the lipid profile is observed. Effectively, the involvement of lipid alterations in AD brains has been well-established in the last years, in particular with reference to PUFA and cholesterol contents (reviewed in [37–39]). Alterations of lipid profile affecting both gross brain lipids and lipid rafts were described in different brain regions affected by AD such as entorhinal cortex, hippocampus, and frontal cortex. In particular, it has been reported abnormally low levels of PUFAn-3 (mainly 22:6n-3) and monoenes, along with lower 20:4n-6 and cholesterol contents, among other lipidomic alterations [39, 40]. Importantly, these changes were exhibited at advanced stage of AD, but also in very early stages of the disease, suggesting that lipid alterations are early events in the pathogenesis of AD. Concomitantly, an increased level of lipoxidative damage specifically targeted to proteins involved in energy metabolism, cytoskeleton, neurotransmission, proteostasis, and oxygen metabolism has been described in AD [39]. Interestingly, these affected biological processes play a central role in the neuronal lost and subsequent functional decline associated with AD [39]. Thus, it can be hypothesized that a combination of increased oxidative stress, deficits in mitochondrial bioenergetics, and disruption of lipid homeostasis overcomes the ability to maintain lipid membrane composition, becoming a seminal condition during the development of the disease, in contrast to what occurs during normal brain aging.

Another interesting observation of the present study is that some of the observed changes in fatty acid profile with age showed a breakpoint after the age of 50. This finding is in line with additional changes such as transcriptional defects related to mitochondrial electron transport chain and signaling pathways involved in neuronal survival, decreased concentration of the main lipid classes, and increased oxidation-derived protein damage that also take place at this age [38, 41–43]. In this context, it is plausible to postulate that these adaptive changes might represent, when they reach a threshold value, the molecular substrate determining a bifurcation of the normal temporal trajectory toward the onset of AD pathology.

Globally, age-associated changes in terms of individual fatty acid content in the brain are heterogeneous. Thus, forebrain appears to be the region most affected by the aging process. Few changes were found in substantia nigra in the midbrain, whereas hindbrain global fatty acid composition appeared to be unaffected by the aging process. Our findings also suggest that major adult human brain fatty acids undergo slight but progressive and significant changes in their abundance during the aging process, with some changes showing a breakpoint after the age of 50. However, the individual contribution of these fatty acid patterns to the aging process is as yet unknown. Therefore, goals of future research are to define which types of lipid molecular species change with age in the different human brain regions, to extend the lipidomics analysis to additional brain regions not studied, to analyze lipid patterns according to neural cell-type specificity, and how they relate both to the function of the area and to the dysfunction leading to neuropathology. Indeed, it is not yet known whether the changes in fatty acids represent neutral changes with age, changes causing physiological aspects of aging, or adaptative responses to damaging agents. In any case, the findings described here suggest that fatty acids and their metabolism are closely linked to human brain aging.

## Supplementary Information

Below is the link to the electronic supplementary material.

Supplementary file1 (DOCX 86 KB)

## Data Availability

The datasets generated and/or analyzed during the current study are available from the corresponding author upon reasonable request.
