# Supplementary material for: Selective brain regional changes in lipid profile with human aging
Source: GeroScience. 2022 Feb 11;44(2):763–83. doi: 10.1007/s11357-022-00527-1 (PMC9135931; doi:10.1007/s11357-022-00527-1)
Supplement: Supplementary file 1 — Supplementary file1 (DOCX 86 KB) [file 11357_2022_527_MOESM1_ESM.docx]

**Selective Brain Regional Changes in Lipid Profile with Human Aging**

**Natalia Mota-Martorell ^1^, Pol Andrés-Benito ^2^, Meritxell Martín-Gari ^1^, José Daniel Galo-Licona ^1^, Joaquim Sol ^1^, Anna Fernández-Bernal ^1^, Manuel Portero-Otín ^1^, Isidro Ferrer ^2,3,4^, Mariona Jove ^1,*^, Reinald Pamplona ^1,*^**

^1^ Department of Experimental Medicine, University of Lleida—Lleida Biomedical Research Institute (UdL-IRBLleida), E-25198 Lleida, Spain; [nataliamotamartorell@gmail.com](mailto:nataliamotamartorell@gmail.com) (N.M-M.); [meritxell.martin@udl.cat](mailto:meritxell.martin@udl.cat) (M.M-G.); [jgalolic25@gmail.com](mailto:jgalolic25@gmail.com) (J.D.G-L.); [solcullere@gmail.com](mailto:solcullere@gmail.com) (J.S.); [anna.fernandez@udl.cat](mailto:anna.fernandez@udl.cat) (A.F.); [manuel.portero@udl.cat](mailto:manuel.portero@udl.cat) (M.P.-O.); [mariona.jove@udl.cat](mailto:mariona.jove@udl.cat) (M.J.); [reinald.pamplona@udl.cat](mailto:reinald.pamplona@udl.cat) (R.P.)

^2^ Center for Biomedical Research on Neurodegenerative Diseases (CIBERNED), Institute of Health Carlos III, E-28220 Madrid, Spain; [pol.andres.benito@gmail.com](mailto:pol.andres.benito@gmail.com) (P.A-B.); [8082ifa@gmail.com](mailto:8082ifa@gmail.com) (I.F.)

^3^ Department of Pathology and Experimental Therapeutics, University of Barcelona, E-08907 L’Hospitalet de Llobregat, Barcelona, Spain; [8082ifa@gmail.com](mailto:8082ifa@gmail.com) (I.F.)

^4^ Emerito researcher, Institute of Biomedical Research of Bellvitge (IDIBELL), Hospitalet de Llobregat, Spain; [8082ifa@gmail.com](mailto:8082ifa@gmail.com) (I.F.)

*Correspondence: [mariona.jove@udl.cat](mailto:mariona.jove@udl.cat) (M.J.); [reinald.pamplona@udl.cat](mailto:reinald.pamplona@udl.cat) (R.P.)

***Supplementary Table* *S1*. Estimated elongase (Elovl) and desaturase (Delta-X-desaturase, DXD) activity of the different brain regions analyzed comparing middle-aged and elderly groups.** Healthy individuals were grouped within middle aged (<60 yrs) and the elderly (>60 yrs). Differences between groups were assessed by applying a t-test and corrected by FDR method of Benhamini and Hochberg, with Q=10. Concentration values are reported in mol% as mean ± SEM from 8-9 individuals x group. P < 0.05 was selected as the minimum level of statistical significance. Significance prior FDR correction is denoted using asterisks (*P<0.05; **P<0.01; ***P<0.001).

|  | **HINDBRAIN** | | | | | | **MIDBRAIN** | | |
| --- | --- | --- | --- | --- | --- | --- | --- | --- | --- |
|  |  |  |  |  |  |  |  |  |  |
|  | **Olive** | | | **Vermis** | | | **Substantia nigra** | | |
|  | ***Middle-aged*** | ***Elderly*** | ***Sig.*** | ***Middle-aged*** | ***Elderly*** | ***Sig.*** | ***Middle-aged*** | ***Elderly*** | ***Sig.*** |
| **D9D (n-7)** | 0.14±0.00 | 0.13±0.01 | 0.597 | 0.05±0.00 | 0.04±0.00 | 0.810 | 0.07±0.00 | 0.07±0.00 | 0.571 |
| **D9D (n-9)** | 1.53±0.05 | 1.62±0.16 | 0.597 | 1.03±0.03 | 0.98±0.05 | 0.803 | 1.06±0.01 | 1.04±0.05 | 0.746 |
| **D5D** | 2.89±0.19 | 3.59±0.99 | 0.574 | 8.25±0.64 | 9.07±0.85 | 0.803 | 4.97±0.30 | 5.94±0.89 | 0.501 |
| **D6D (a)** | 0.27±0.04 | 0.19±0.02 | 0.726 | 0.47±0.02 | 0.45±0.01 | 0.803 | 0.19±0.01 | 0.39±0.06* | 0.155 |
| **D6D (b)** | 6.13±0.64 | 6.14±0.53 | >0.999 | 2.93±0.50 | 2.42±0.19 | 0.803 | 4.57±0.51 | 2.92±0.28* | 0.173 |
| **Elovl6** | 1.31±0.05 | 1.22±0.02 | 0.597 | 0.83±0.01 | 0.84±0.03 | 0.813 | 1.28±0.01 | 1.26±0.03 | 0.616 |
| **Elovl1-3-7 (a)** | 0.02±0.00 | 0.02±0.00 | 0.814 | 0.011±0.00 | 0.013±0.00** | 0.068 | 0.01±0.00 | 0.01±0.00 | 0.821 |
| **Elovl1-3-7 (b)** | 0.82±0.04 | 0.82±0.04 | >0.999 | 0.29±0.01 | 0.25±0.01* | 0.763 | 0.53±0.03 | 0.46±0.03 | 0.571 |
| **Elovl1-3-7 (c)** | 3.44±0.27 | 3.37±0.54 | 0.939 | 1.59±0.17 | 1.35±0.13 | 0.803 | 0.34±0.05 | 1.77±0.33** | 0.049 |
| **Elovl3 (n-9) (a)** | 0.14±0.00 | 0.13±0.01 | 0.726 | 0.06±0.00 | 0.06±0.00 | 0.872 | 0.09±0.00 | 0.08±0.00 | 0.571 |
| **Elovl3 (n-9) (b)** | 0.18±0.02 | 0.17±0.79 | 0.939 | 0.64±0.07 | 0.68±0.08 | 0.906 | 0.46±0.11 | 0.39±0.33 | 0.746 |
| **Elovl3 (n-9) (c)** | 4.70±0.63 | 4.99±0.86 | 0.909 | 0.67±0.12 | 0.56±0.08 | 0.813 | 1.98±0.26 | 1.87±0.34 | 0.821 |
| **Elovl5** | 1.82±0.37 | 1.87±0.31 | 0.981 | 0.36±0.07 | 0.29±0.03 | 0.803 | 0.69±0.10 | 0.59±0.06 | 0.657 |
| **Elovl2-5 (n-6)** | 0.87±0.03 | 0.84±0.05 | 0.775 | 0.36±0.01 | 0.35±0.02 | 0.813 | 0.67±0.01 | 0.63±0.03 | 0.571 |
| **Elovl2-5 (n-3)** | 5.88±1.29 | 6.99±1.32 | 0.775 | 7.76±1.02 | 8.55±0.95 | 0.813 | 3.53±0.28 | 4.20±0.54 | 0.571 |
| **Elovl2** | 0.43±0.02 | 0.41±0.07 | 0.788 | 0.20±0.02 | 0.19±0.02 | 0.944 | 0.21±0.02 | 0.27±0.02* | 0.183 |

**Table S1. Cont.**

|  | **FOREBRAIN** | | | | | | | | | | | |
| --- | --- | --- | --- | --- | --- | --- | --- | --- | --- | --- | --- | --- |
|  | **DIENCEPHALON** | | | **SUBCORTICAL TELENCEPHALON** | | | | | | | | |
|  | **Thalamus** | | | **Hippocampus** | | | **Caudate** | | | **Putamen** | | |
|  | ***Middle-aged*** | ***Elderly*** | ***Sig.*** | ***Middle-aged*** | ***Elderly*** | ***Sig.*** | ***Middle-aged*** | ***Elderly*** | ***Sig.*** | ***Middle-aged*** | ***Elderly*** | ***Sig.*** |
| **D9D (n-7)** | 0.07±0.00 | 0.06±0.00 | 0.687 | 0.06±0.00 | 0.05±0.01 | 0.759 | 0.04±0.00 | 0.04±0.00 | 0.673 | 0.04±0.00 | 0.05±0.00 | 0.102 |
| **D9D (n-9)** | 0.99±0.04 | 1.05±0.09 | 0.796 | 0.95±0.04 | 0.90±0.07 | 0.759 | 0.68±0.02 | 0.66±0.06 | 0.671 | 0.77±0.02 | 0.84±0.05 | 0.156 |
| **D5D** | 8.93±0.72 | 11.29±2.00 | 0.687 | 7.50±0.84 | 8.04±0.80 | 0.759 | 9.22±0.63 | 10.38±0.89 | 0.587 | 9.36±0.65 | 10.93±0.95 | 0.177 |
| **D6D (a)** | 0.29±0.01 | 0.46±0.12 | 0.675 | 0.34±0.03 | 0.39±0.04 | 0.759 | 0.72±0.08 | 0.76±0.10 | 0.874 | 0.26±0.02 | 0.28±0.05 | 0.638 |
| **D6D (b)** | 5.98±0.70 | 3.74±0.47* | 0.401 | 6.13±0.50 | 4.94±0.56 | 0.759 | 2.38±0.29 | 1.64±0.27 | 0.270 | 6.70±0.95 | 4.76±0.55 | 0.126 |
| **Elovl6** | 1.30±0.04 | 1.19±0.03 | 0.687 | 1.05±0.03 | 1.03±0.02 | 0.759 | 1.18±0.02 | 1.19±0.06 | 0.935 | 1.24±0.03 | 1.16±0.02 | 0.134 |
| **Elovl1-3-7 (a)** | 0.01±0.00 | 0.01±0.00 | 0.687 | 0.01±0.00 | 0.01±0.00 | 0.805 | 0.007±0.00 | 0.008±0.00* | 0.195 | 0.01±0.00 | 0.01±0.00 | 0.087 |
| **Elovl1-3-7 (b)** | 0.53±0.05 | 0.48±0.04 | 0.796 | 0.63±0.05 | 0.58±0.02 | 0.759 | 0.47±0.02 | 0.36±0.02** | 0.195 | 0.55±0.03 | 0.45±0.04* | 0.026 |
| **Elovl1-3-7 (c)** | 2.93±0.20 | 2.36±0.38 | 0.687 | 0.39±0.09 | 0.32±0.35 | 0.759 | 2.12±0.27 | 1.22±0.13* | 0.195 | 0.17±0.03 | 0.30±0.11* | 0.022 |
| **Elovl3 (n-9) (a)** | 0.07±0.00 | 0.06±0.00 | 0.687 | 0.04±0.00 | 0.03±0.01 | 0.759 | 0.04±0.00 | 0.03±0.00* | 0.249 | 0.05±0.00 | 0.05±0.00 | 0.299 |
| **Elovl3 (n-9) (b)** | 0.65±0.03 | 0.64±0.26 | 0.937 | 1.03±0.20 | 0.79±0.12 | 0.759 | 1.66±0.17 | 2.05±0.31 | 0.535 | 0.95±0.04 | 0.46±0.34 | 0.376 |
| **Elovl3 (n-9) (c)** | 2.24±0.39 | 1.57±0.39 | 0.687 | 1.73±0.54 | 1.65±0.19 | 0.891 | 0.53±0.07 | 0.38±0.13 | 0.535 | 2.31±0.23 | 3.00±0.82 | 0.424 |
| **Elovl5** | 0.58±0.06 | 0.45±0.08 | 0.687 | 0.80±0.21 | 0.59±0.16 | 0.759 | 0.18±0.02 | 0.15±0.03 | 0.601 | 0.21±0.02 | 0.23±0.04 | 0.705 |
| **Elovl2-5 (n-6)** | 0.67±0.04 | 0.52±0.05* | 0.542 | 0.62±0.04 | 0.54±0.03 | 0.759 | 0.51±0.02 | 0.44±0.02* | 0.219 | 0.57±0.01 | 0.50±0.04 | 0.080 |
| **Elovl2-5 (n-3)** | 3.85±0.53 | 4.26±0.38 | 0.937 | 10.55±1.90 | 8.61±1.09 | 0.759 | 4.45±0.47 | 4.16±0.50 | 0.839 | 6.17±0.86 | 5.86±0.92 | 0.817 |
| **Elovl2** | 0.16±0.01 | 0.18±0.02 | 0.694 | 0.14±0.01 | 0.13±0.06 | 0.826 | 0.22±0.02 | 0.19±0.02 | 0.601 | 0.13±0.01 | 0.18±0.01* | 0.021 |

**Table S1. Cont.**

|  | **FOREBRAIN** | | | | | | | | |
| --- | --- | --- | --- | --- | --- | --- | --- | --- | --- |
|  | **CORTICAL TELENCEPHALON** | | | | | | | | |
|  | **Occipital cortex** | | | **Parietal cortex** | | | **Temporal cortex** | | |
|  | ***Middle-aged*** | ***Elderly*** | ***Sig.*** | ***Middle-aged*** | ***Elderly*** | ***Sig.*** | ***Middle-aged*** | ***Elderly*** | ***Sig.*** |
| **D9D (n-7)** | 0.05±0.00 | 0.04±0.00 | 0.825 | 0.06±0.00 | 0.05±0.00 | 0.846 | 0.04±0.002 | 0.03±0.00 | 0.940 |
| **D9D (n-9)** | 0.73±0.03 | 0.78±0.05 | 0.793 | 0.81±0.04 | 0.81±0.03 | 0.973 | 0.609±0.021 | 0.64±0.02 | 0.470 |
| **D5D** | 7.55±0.58 | 7.96±0.84 | 0.982 | 7.34±0.66 | 8.16±0.86 | 0.846 | 9.185±0.631 | 9.28±0.80 | 0.971 |
| **D6D (a)** | 0.48±0.06 | 0.65±0.12 | 0.592 | 0.49±0.06 | 0.57±0.10 | 0.863 | 0.801±0.058 | 0.74±0.08 | 0.773 |
| **D6D (b)** | 4.51±0.43 | 5.15±0.97 | 0.982 | 4.16±0.72 | 3.82±0.49 | 0.973 | 3.338±0.325 | 2.17±1.37* | 0.147 |
| **Elovl6** | 1.13±0.01 | 1.12±0.02 | 0.982 | 1.15±0.02 | 1.18±0.02 | 0.846 | 1.00±0.01 | 1.042±0.048** | 0.202 |
| **Elovl1-3-7 (a)** | 0.008±0.00 | 0.009±0.00* | 0.494 | 0.01±0.00 | 0.01±0.00 | 0.973 | 0.01±0.00 | 0.007±0** | 0.173 |
| **Elovl1-3-7 (b)** | 0.49±0.02 | 0.47±0.05 | 0.982 | 0.50±0.03 | 0.51±0.05 | 0.973 | 0.322±0.015 | 0.30±0.04 | 0.773 |
| **Elovl1-3-7 (c)** | 1.35±0.15 | 1.22±0.22 | 0.982 | 2.05±0.20 | 1.87±0.24 | 0.876 | 0.863±0.095 | 1.00±0.25 | 0.773 |
| **Elovl3 (n-9) (a)** | 0.05±0.00 | 0.05±0.00 | 0.982 | 0.05±0.00 | 0.05±0.00 | 0.846 | 0.03±0.003 | 0.03±0.00 | 0.773 |
| **Elovl3 (n-9) (b)** | 2.46±0.32 | 2.35±0.33 | 0.982 | 0.83±0.12 | 0.52±0.08 | 0.846 | 2.059±0.538 | 1.87±0.15 | 0.896 |
| **Elovl3 (n-9) (c)** | 0.38±0.06 | 0.40±0.15 | 0.982 | 1.94±0.49 | 2.00±0.52 | 0.973 | 0.327±0.124 | 0.43±0.08 | 0.773 |
| **Elovl5** | 0.29±0.04 | 0.20±0.04 | 0.705 | 0.29±0.04 | 0.23±0.03 | 0.846 | 0.198±0.039 | 0.16±0.08 | 0.773 |
| **Elovl2-5 (n-6)** | 0.53±0.01 | 0.48±0.02 | 0.592 | 0.54±0.03 | 0.49±0.03 | 0.846 | 0.447±0.012 | 0.44±0.04 | 0.971 |
| **Elovl2-5 (n-3)** | 6.39±0.64 | 6.32±0.68 | 0.982 | 6.77±0.91 | 6.39±0.72 | 0.973 | 5.763±0.842 | 5.90±0.61 | 0.971 |
| **Elovl2** | 0.12±0.01 | 0.09±0.01 | 0.494 | 0.16±0.00 | 0.20±0.04 | 0.846 | 0.164±0.012 | 0.11±0.01* | 0.147 |

**Table S1. Cont.**

|  | **FOREBRAIN** | | | | | | | | |
| --- | --- | --- | --- | --- | --- | --- | --- | --- | --- |
|  | **CORTICAL TELENCEPHALON** | | | | | | | | |
|  | **Entorhinal cortex** | | | **Frontal cortex** | | | **Cingulate cortex** | | |
|  | ***Middle-aged*** | ***Elderly*** | ***Sig.*** | ***Middle-aged*** | ***Elderly*** | ***Sig.*** | ***Middle-aged*** | ***Elderly*** | ***Sig.*** |
| **D9D (n-7)** | 0.06±0.00 | 0.07±0.01 | 0.691 | 0.06±0.00 | 0.05±0.00 | 0.953 | 0.06±0.01 | 0.07±0.00 | 0.934 |
| **D9D (n-9)** | 0.77±0.04 | 0.88±0.06 | 0.691 | 0.74±0.04 | 0.74±0.03 | 0.964 | 0.82±0.07 | 0.87±0.05 | 0.934 |
| **D5D** | 9.36±0.98 | 8.72±0.88 | 0.823 | 8.13±0.50 | 9.15±0.90 | 0.953 | 8.29±0.53 | 8.21±0.60 | 0.970 |
| **D6D (a)** | 0.57±0.09 | 0.48±0.04 | 0.705 | 0.65±0.05 | 0.62±0.09 | 0.964 | 0.57±0.08 | 0.48±0.05 | 0.934 |
| **D6D (b)** | 4.20±0.82 | 4.53±0.69 | 0.888 | 2.93±0.43 | 2.89±0.39 | 0.964 | 3.89±1.17 | 4.07±0.73 | 0.970 |
| **Elovl6** | 1.16±0.03 | 1.19±0.03 | 0.759 | 1.10±0.01 | 1.10±0.03 | 0.968 | 0.97±0.03 | 1.00±0.04 | 0.934 |
| **Elovl1-3-7 (a)** | 0.009±0.00 | 0.01±0.00 | 0.691 | 0.01±0.00 | 0.01±0.00 | 0.953 | 0.008±0.00 | 0.01±0.00* | 0.376 |
| **Elovl1-3-7 (b)** | 0.42±0.04 | 0.50±0.05 | 0.691 | 0.41±0.04 | 0.39±0.03 | 0.964 | 0.44±0.06 | 0.42±0.04 | 0.952 |
| **Elovl1-3-7 (c)** | 2.25±0.23 | 2.64±0.29 | 0.695 | 1.84±0.24 | 1.81±0.22 | 0.964 | 1.96±0.29 | 2.12±0.24 | 0.934 |
| **Elovl3 (n-9) (a)** | 0.04±0.00 | 0.04±0.00 | 0.763 | 0.04±0.00 | 0.04±0.00 | 0.953 | 0.04±0.00 | 0.05±0.00 | 0.934 |
| **Elovl3 (n-9) (b)** | 0.98±0.16 | 0.68±0.15 | 0.691 | 1.45±0.26 | 1.42±0.23 | 0.964 | 0.98±0.12 | 0.82±0.16 | 0.934 |
| **Elovl3 (n-9) (c)** | 1.29±0.32 | 2.20±0.57 | 0.691 | 0.73±0.31 | 0.62±0.16 | 0.964 | 1.12±0.51 | 1.38±0.44 | 0.934 |
| **Elovl5** | 0.45±0.08 | 0.43±0.08 | 0.908 | 0.22±0.02 | 0.19±0.07 | 0.953 | 0.50±0.16 | 0.39±0.12 | 0.934 |
| **Elovl2-5 (n-6)** | 0.56±0.03 | 0.59±0.03 | 0.823 | 0.49±0.03 | 0.45±0.02 | 0.953 | 0.59±0.04 | 0.58±0.03 | 0.970 |
| **Elovl2-5 (n-3)** | 6.12±0.69 | 5.86±0.65 | 0.890 | 4.92±0.50 | 6.44±0.51 | 0.953 | 6.51±0.90 | 6.99±1.03 | 0.934 |
| **Elovl2** | 0.14±0.01 | 0.16±0.01 | 0.759 | 0.16±0.02 | 0.12±0.01 | 0.953 | 0.15±0.01 | 0.14±0.01 | 0.934 |

Elongase and desaturase activity was estimated from specific product/substrate ratios. For desaturase activity: D9D (n-7) = 16:1n-9/16:0; D9D (n-9) = 18:1n-9/18:0; D5D (n-6) = 20:4n-6/20:3n-6; D6D (n-3) (a) = 18:4n-3/18:3n-3; D6D (n-3) (b) = 24:6n-3/24:5n-3. For elongase activity: Elovl3 (n-9) (a) = 20:1n-9/18:1n-9; Elovl3 (n-9) (b) = 22:1n-9/20:1n-9; Elovl3 (n-9) (c) = 24:1n-9/22:1n-9; Elovl6 = 18:0/16:0; Elovl1-3-7 (a) = 20:0/18:0; Elovl1-3-7 (b) = 22:0/20:0; Elovl1-3-7 (c) = 24:0/22:0; Elovl 5(n-6) = 20:2n-6/18:2n-6; Elovl2-5 (n-6) = 22:4n-6/20:4n-6; Elovl 2-5(n-3) = 22:5n-3/20:5n-3, and Elovl 2(n-3) = 24:5n-3/22:5n-3.

**Supplementary Table S2. Correlations between individual fatty acids and calculated indexes with age in 13 different regions of the human brain.** Spearman correlation was performed between age and mol% for each individual fatty acid and measured index and corrected by FDR method of Benhamini and Hochberg, with Q=10. Significance prior FDR correction is denoted using asterisks (*P<0.05; **P<0.01; ***P<0.001).

|  | Hindbrain | | Midbrain | Forebrain | | | | | | | | | |
| --- | --- | --- | --- | --- | --- | --- | --- | --- | --- | --- | --- | --- | --- |
|  |  |  |  | Subcortical telencephalon | | | | Cortical telencephalon | | | | | |
|  | Vermis | Olive | Substantia  nigra | Thalamus | Hippocampus | Caudate | Putamen | Occipital  cortex | Parietal  cortex | Temporal  cortex | Entorhinal  cortex | Frontal  cortex | Cingulate  cortex |
| 14:0 | -0.13  (p=0.97) | -0.8  (p<0.001)*** | 0.28  (p=0.556) | -0.34  (p=0.697) | -0.38  (p=0.753) | -0.03  (p=0.978) | 0.2  (p=0.599) | -0.29  (p=0.675) | -0.54  (p=0.135)* | 0.46  (p=0.178) | 0.15  (p=0.679) | 0.03  (p=0.94) | 0.04  (p=0.909) |
| 16:0 | -0.01  (p=0.983) | 0.19  (p=0.704) | 0.19  (p=0.706) | 0.33  (p=0.697) | 0.14  (p=0.753) | 0.09  (p=0.86) | 0.55  (p=0.154)* | -0.22  (p=0.68) | -0.12  (p=0.859) | -0.13  (p=0.803) | -0.3  (p=0.338) | -0.22  (p=0.747) | -0.63  (p=0.045)* |
| 16:1n7 | 0.03  (p=0.983) | -0.36  (p=0.396) | 0.55  (p=0.169)* | -0.01  (p=0.985) | -0.14  (p=0.753) | -0.24  (p=0.614) | 0.4  (p=0.399) | -0.21  (p=0.68)* | -0.36  (p=0.493) | -0.05  (p=0.891) | 0.47  (p=0.271) | 0.03  (p=0.94) | 0.56  (p=0.059)* |
| 18:0 | 0.08  (p=0.97) | -0.5  (p=0.277) | 0.38  (p=0.414) | -0.02  (p=0.985) | 0.17  (p=0.753) | 0.12  (p=0.86) | -0.2  (p=0.533) | -0.6  (p=0.415) | -0.04  (p=0.968) | 0.25  (p=0.525) | -0.41  (p=0.271) | -0.38  (p=0.53) | -0.32  p=0.407) |
| 18:1n9 | -0.08  (p=0.97) | 0.4  (p=0.338) | -0.02  (p=0.953) | -0.15  (p=0.793) | -0.04  (p=0.885) | -0.23  (p=0.614) | 0.1  (p=0.657) | 0.21  (p=0.68) | 0.01  (p=0.97) | 0.71  (p=0.018)** | 0.42  (p=0.271) | 0.33  (p=0.53) | 0.56  p=0.059)* |
| 18:1n7 | -0.09  (p=0.97) | 0.01  (p=0.96) | 0.11  (p=0.817) | 0.24  (p=0.697) | 0.08  (p=0.8) | 0.0  (p=0.996) | 0.2  (p=0.533) | 0.2  (p=0.68) | 0.14  (p=0.859) | 0.35  (p=0.348) | 0.35  (p=0.313) | 0.2  (p=0.773) | 0.29  p=0.474) |
| 18:2n6 | -0.02  (p=0.983) | -0.08  (p=0.865) | -0.23  (p=0.619) | 0.09  (p=0.882) | 0.1  (p=0.8) | -0.14  (p=0.86) | -0.26  (p=0.533) | 0.07  (p=0.904) | -0.07  (p=0.912) | 0.16  (p=0.743) | -0.11  (p=0.725) | -0.28  (p=0.65) | -0.14  (p=0.761) |
| 18:3n3 | 0.0  (p=0.996) | -0.21  (p=0.656) | 0.04  (p=0.953) | -0.34  (p=0.697) | -0.2  (p=0.753) | -0.58  (p=0.191)* | -0.43  (p=0.354) | 0.17  (p=0.735) | -0.48  (p=0.223) | 0.13  (p=0.803) | 0.32  (p=0.328) | -0.11  (p=0.876) | 0.37  (p=0.305) |
| 18:4n3 | -0.22  (p=0.97) | -0.22  (p=0.644) | 0.65  (p=0.105)** | 0.39  (p=0.697) | 0.21  (p=0.753) | -0.32  (p=0.587) | 0.27  (p=0.533) | 0.41  (p=0.438) | -0.32  (p=0.618) | -0.02  (p=0.94) | 0.23  (p=0.494) | -0.34  (p=0.53) | 0.25  (p=0.542) |
| 20:0 | 0.75  (p=0.035)** | 0.12  (p=0.82) | 0.44  (p=0.27) | 0.09  (p=0.882) | 0.14  (p=0.753) | 0.5  (p=0.191)* | 0.14  (p=0.657) | 0.32  (p=0.624) | -0.13  (p=0.859) | 0.39  (p=0.308) | 0.59  (p=0.271)* | 0.56  (p=0.403)* | 0.7  (p=0.045)** |
| 20:1n9 | 0.09  (p=0.97) | -0.43  (p=0.285) | 0.11  (p=0.817) | -0.24  (p=0.697) | -0.28  (p=0.753) | -0.53  (p=0.191)* | -0.32  (p=0.513) | 0.12  (p=0.812) | -0.1  (p=0.894) | 0.25  (p=0.525) | 0.33  (p=0.319) | -0.06  (p=0.94) | 0.52  (p=0.082)* |
| 20:2n6 | -0.04  (p=0.983) | 0.11  (p=0.82) | -0.03  (p=0.953) | -0.12  (p=0.85) | -0.21  (p=0.753) | -0.37  (p=0.465) | -0.22  (p=0.599) | 0.03  (p=0.978) | -0.18  (p=0.798) | -0.07  (p=0.891) | 0.36  (p=0.313) | -0.12  (p=0.876) | 0.16  (p=0.759) |
| 20:3n6 | -0.21  (p=0.97) | -0.23  (p=0.644) | -0.19  (p=0.706) | -0.01  (p=0.985) | -0.29  (p=0.753) | 0.08  (p=0.86) | -0.14  (p=0.657) | 0.12  (p=0.812) | -0.01  (p=0.97) | -0.26  (p=0.525) | -0.17  (p=0.636) | -0.28  (p=0.65) | 0.06  (p=0.888) |
| 20:4n6 | -0.23  (p=0.97) | 0.48  (p=0.277) | -0.06  (p=0.921) | 0.17  (p=0.793) | 0.14  (p=0.753) | 0.49  (p=0.191)* | 0.33  (p=0.513) | 0.01  (p=0.981) | 0.22  (p=0.709) | -0.52  (p=0.119)* | -0.43  (p=0.271) | -0.14  (p=0.864) | -0.56  (p=0.059)* |
| 20:5n3 | -0.08  (p=0.97) | 0.15  (p=0.786) | -0.26  (p=0.586) | 0.01  (p=0.985) | 0.14  (p=0.753) | 0.25  (p=0.614) | 0.18  (p=0.645) | 0.23  (p=0.68) | 0.24  (p=0.709) | 0.08  (p=0.891) | 0.12  (p=0.718) | -0.07  (p=0.94) | 0.1  (p=0.817) |
| 22:0 | 0.3  (p=0.97) | 0.31  (p=0.521) | -0.03  (p=0.953) | -0.23  (p=0.697) | -0.04  (p=0.885) | -0.41  (p=0.357) | -0.37  (p=0.461) | 0.25  (p=0.68) | -0.11  (p=0.859) | 0.24  (p=0.525) | 0.45  (p=0.271) | 0.14  (p=0.864) | 0.56  (p=0.059)* |
| 22:1n9 | 0.23  (p=0.97) | -0.25  (p=0.632) | -0.11  (p=0.817) | 0.02  (p=0.985) | -0.51  (p=0.42)* | 0.01  (p=0.996) | -0.42  (p=0.354) | 0.05  (p=0.932) | -0.27  (p=0.709) | -0.36  (p=0.335) | -0.35  (p=0.313) | -0.35  (p=0.53) | -0.06  (p=0.888) |
| 22:4n6 | -0.34  (p=0.97) | 0.25  (p=0.632) | -0.47  (p=0.245) | -0.86  (p=<0.001)*** | -0.56  (p=0.42)* | -0.63  (p=0.191)** | -0.81  (p<0.001)*** | -0.46  (p=0.415) | -0.23  (p=0.709) | -0.69  (p=0.023)** | -0.12  (p=0.718) | -0.36  (p=0.53) | -0.25  (p=0.542) |
| 22:5n6 | -0.17  (p=0.97) | -0.06  (p=0.895) | -0.46  (p=0.256) | -0.32  (p=0.697) | -0.14  (p=0.753) | -0.48  (p=0.191)* | -0.65  (p=0.053)** | -0.38  (p=0.537) | -0.27  (p=0.709) | -0.64  (p=0.053)** | -0.47  (p=0.271) | -0.58  (p=0.403)* | -0.58  (p=0.059)* |
| 22:5n3 | -0.37  (p=0.97) | 0.62  (p=0.198)* | -0.14  (p=0.8) | 0.58  (p=0.268)* | 0.28  (p=0.753) | 0.2  (p=0.663) | 0.2  (p=0.619) | 0.47  (p=0.415) | 0.44  (p=0.277) | 0.12  (p=0.803) | 0.07  (p=0.809) | 0.33  (p=0.53) | 0.2  (p=0.652) |
| 22:6n3 | 0.17  (p=0.97) | 0.29  (p=0.562) | -0.33  (p=0.461) | 0.44  (p=0.572) | 0.48  (p=0.464) | 0.08  (p=0.86) | 0.17  (p=0.645) | 0.25  (p=0.68) | 0.63  (p=0.061)** | 0.31  (p=0.43) | -0.33  (p=0.319) | 0.43  (p=0.53) | 0.12  (p=0.77) |
| 24:0 | 0.07  (p=0.97) | 0.06  (p=0.895) | 0.72  (p=0.07)** | -0.19  (p=0.746) | 0.2  (p=0.753) | -0.49  (p=0.191)* | 0.5  (p=0.239)* | -0.01  (p=0.981) | -0.17  (p=0.821) | 0.21  (p=0.602) | 0.4  (p=0.271) | 0.21  (p=0.747) | 0.57  (p=0.059)* |
| 24:1n9 | 0.12  (p=0.97) | -0.14  (p=0.786) | -0.1  (p=0.817) | -0.3  (p=0.697) | -0.17  (p=0.753) | -0.52  (p=0.191)* | -0.35  (p=0.484) | 0.08  (p=0.883) | -0.05  (p=0.962) | 0.38  (p=0.315) | 0.42  (p=0.271) | 0.14  (p=0.864) | 0.58  (p=0.059)* |
| 24:5n3 | -0.07  (p=0.97) | 0.26  (p=0.632) | 0.48  (p=0.245) | 0.47  (p=0.525) | 0.29  (p=0.753) | -0.22  (p=0.614) | 0.64  (p=0.053)** | -0.24  (p=0.68) | 0.44  (p=0.277) | -0.55  (p=0.105)* | 0.45  (p=0.271) | 0.06  (p=0.94) | 0.12  (p=0.77) |
| 24:6n3 | -0.18  (p=0.97) | 0.61  (p=0.198)* | -0.24  (p=0.619) | -0.22  (p=0.697) | -0.2  (p=0.753) | -0.57  (p=0.191)* | -0.21  (p=0.599) | 0.17  (p=0.735) | -0.03  (p=0.97) | -0.6  (p=0.077)* | 0.3  (p=0.338) | 0.04  (p=0.94) | 0.58  (p=0.059)* |
| ACL | -0.14  (p=0.97) | 0.11  (p=0.82) | -0.58  (p=0.169)* | -0.82  (p<0.001)*** | -0.15  (p=0.753) | -0.27  (p=0.614) | -0.81  (p<0.001)*** | 0.43  (p=0.415) | 0.63  (p=0.061)** | -0.5  (p=0.127)* | 0.04  (p=0.888) | 0.16  (p=0.864) | 0.63  (p=0.045)** |
| SFA | 0.16  (p=0.97) | -0.44  (p=0.28) | 0.32  (p=0.461) | 0.23  (p=0.697) | 0.09  (p=0.8) | 0.07  (p=0.86) | 0.26  (p=0.533) | -0.44  (p=0.415) | -0.22  (p=0.709) | -0.04  (p=0.891) | -0.41  (p=0.271) | -0.34  (p=0.53) | -0.64  (p=0.045)** |
| UFA | -0.16  (p=0.97) | 0.44  (p=0.28) | -0.32  (p=0.461) | -0.23  (p=0.697) | -0.09  (p=0.8) | -0.07  (p=0.86) | -0.26  (p=0.533) | 0.44  (p=0.415) | 0.22  (p=0.709) | 0.04  (p=0.891) | 0.41  (p=0.271) | 0.34  (p=0.53) | 0.64  (p=0.045)** |
| MUFA | -0.02  (p=0.983) | -0.01  (p=0.96) | 0.17  (p=0.743) | -0.22  (p=0.697) | -0.2  (p=0.753) | -0.3  (p=0.608) | -0.13  (p=0.657) | 0.11  (p=0.812) | -0.08  (p=0.912) | 0.56  (p=0.105)* | 0.46  (p=0.271) | 0.22  (p=0.747) | 0.55  (p=0.059)* |
| PUFA | -0.21  (p=0.97) | 0.5  (p=0.277) | -0.56  (p=0.169)* | 0.13  (p=0.827) | 0.21  (p=0.753) | 0.23  (p=0.614) | -0.15  (p=0.657) | 0.11  (p=0.812) | 0.58  (p=0.105)* | -0.55  (p=0.105)* | -0.43  (p=0.271) | -0.02  (p=0.94) | -0.28  (p=0.495) |
| PUFAn3 | 0.15  (p=0.97) | 0.5  (p=0.277) | -0.31  (p=0.461) | 0.49  (p=0.525) | 0.52  (p=0.42)* | 0.08  (p=0.86) | 0.17  (p=0.645) | 0.32  (p=0.624) | 0.66  (p=0.061)** | 0.25  (p=0.525) | -0.34  (p=0.319) | 0.42  (p=0.53) | 0.17  (p=0.732) |
| PUFAn6 | -0.43  (p=0.97) | 0.5  (p=0.277) | -0.33  (p=0.461) | -0.27  (p=0.697) | -0.17  (p=0.753) | 0.28  (p=0.614) | -0.44  (p=0.354) | -0.36  (p=0.543) | -0.12  (p=0.859) | -0.71  (p=0.018)** | -0.58  (p=0.271)* | -0.45  (p=0.53) | -0.69  (p=0.045)** |
| DBI | -0.23  (p=0.97) | 0.48  (p=0.277) | -0.56  (p=0.169)* | 0.16  (p=0.793) | 0.24  (p=0.753) | 0.25  (p=0.614) | -0.07  (p=0.813) | 0.28  (p=0.675) | 0.63  (p=0.061)** | -0.52  (p=0.119)* | -0.33  (p=0.319) | 0.22  (p=0.747) | -0.03  (p=0.909) |
| PI | -0.08  (p=0.97) | 0.47  (p=0.277) | -0.48  (p=0.245) | 0.27  (p=0.697) | 0.33  (p=0.753) | 0.34  (p=0.537) | 0.0  (p=0.993) | 0.2  (p=0.68) | 0.56  (p=0.111)* | -0.45  (p=0.137)* | -0.36  (p=0.313) | 0.06  (p=0.94) | -0.14  (p=0.761) |
| SFA/UFA | 0.16  (p=0.97) | -0.44  (p=0.28) | 0.32  (p=0.461) | 0.23  (p=0.697) | 0.09  (p=0.8) | 0.07  (p=0.86) | 0.26  (p=0.533) | -0.44  (p=0.415) | -0.22  (p=0.709) | -0.04  (p=0.891) | -0.41  (p=0.271) | -0.34  (p=0.53) | -0.6  (p=0.045)** |

**Table S2. Cont.**

|  | Hindbrain | | Midbrain | Forebrain | | | | | | | | | |
| --- | --- | --- | --- | --- | --- | --- | --- | --- | --- | --- | --- | --- | --- |
|  |  |  |  | Subcortical telencephalon | | | | Cortical telencephalon | | | | | |
|  | Olive | Vermis | Substantia  nigra | Thalamus | Hippocampus | Caudate | Putamen | Occipital  cortex | Parietal  cortex | Temporal  cortex | Entorhinal  cortex | Frontal  cortex | Cingulate  cortex |
| D9D (a) | -0.55  (p=0.363)* | 0.0  (p>0.999) | 0.39  (p=0.464) | -0.2  (p=0.671) | -0.16  (p=0.932) | -0.26  (p=0.544) | 0.22  (p=0.392) | -0.14  (p=0.991) | -0.19  (p=0.978) | -0.01  (p>0.999) | 0.49  (p=0.415) | 0.0  (p>0.999) | 0.68  (p=0.01)** |
| D9D (b) | 0.45  (p=0.363) | -0.19  (p=0.913) | -0.01  (p=0.961) | -0.05  (p=0.856) | -0.03  (p=0.998) | -0.24  (p=0.544) | 0.2  (p=0.416) | 0.24  (p=0.991) | -0.03  (p>0.999) | 0.47  (p=0.36) | 0.4  (p=0.415) | 0.33  (p=0.95) | 0.6  (p=0.019)* |
| D5D | 0.45  (p=0.363) | 0.12  (p=0.913) | 0.12  (p=0.959) | 0.18  (p=0.671) | 0.23  (p=0.932) | 0.24  (p=0.544) | 0.26  (p=0.35) | -0.09  (p=0.991) | 0.18  (p=0.978) | -0.14  (p=0.853) | -0.1  (p=0.753) | 0.19  (p>0.999) | -0.22  (p=0.297) |
| D6D (a) | -0.1  (p=0.96) | -0.37  (p=0.913) | 0.54  (p=0.137)* | 0.53  (p=0.205)* | 0.15  (p=0.932) | 0.23  (p=0.544) | 0.36  (p=0.237) | 0.38  (p=0.644) | 0.19  (p=0.978) | -0.15  (p=0.853) | -0.11  (p=0.739) | -0.2  (p>0.999) | -0.14  (p=0.373) |
| D6D (b) | 0.05  (p>0.999) | 0.0  (p>0.999) | -0.59  (p=0.1)* | -0.74  (p=0.018)** | -0.31  (p=0.932) | -0.39  (p=0.437) | -0.61  (p=0.022)** | 0.35  (p=0.644) | -0.2  (p=0.978) | -0.55  (p=0.218)* | 0.13  (p=0.739) | 0.1  (p>0.999) | 0.51  (p=0.042)* |
| Elovl6 | -0.36  (p=0.621) | 0.2  (p=0.913) | 0.04  (p=0.959) | -0.48  (p=0.264) | -0.08  (p=0.951) | -0.12  (p=0.722) | -0.6  (p=0.022)* | -0.1  (p=0.991) | 0.1  (p=0.99) | 0.37  (p=0.437) | 0.25  (p=0.507) | -0.05  (p>0.999) | 0.57  (p=0.026)* |
| Elovl1-3-7 (a) | 0.19  (p=0.96) | 0.73  (p=0.016)** | 0.31  (p=0.568) | 0.17  (p=0.671) | 0.04  (p=0.998) | 0.34  (p=0.495) | 0.29  (p=0.307) | 0.5  (p=0.644)* | -0.16  (p=0.978) | 0.29  (p=0.677) | 0.68  (p=0.072)** | 0.53  (p=0.574)* | 0.86  (p=0)*** |
| Elovl1-3-7 (b) | 0.14  (p=0.96) | -0.25  (p=0.913) | -0.33  (p=0.568) | -0.35  (p=0.407) | -0.14  (p=0.932) | -0.69  (p=0.045)** | -0.64  (p=0.022)** | -0.02  (p>0.999) | -0.08  (p=0.99) | -0.21  (p=0.853) | 0.35  (p=0.415) | -0.14  (p>0.999) | 0.19  (p=0.326) |
| Elovl1-3-7 (c) | -0.19  (p=0.96) | -0.11  (p=0.913) | 0.72  (p=0.031)** | -0.32  (p=0.436) | 0.11  (p=0.951) | -0.37  (p=0.437) | 0.6  (p=0.022)* | -0.0  (p=0.991) | -0.12  (p=0.978) | 0.13  (p=0.853) | 0.29  (p=0.477) | 0.15  (p>0.999) | 0.67  (p=0.01)** |
| Elovl3 (n-9) (a) | -0.5  (p=0.363) | 0.17  (p=0.913) | 0.05  (p=0.959) | -0.38  (p=0.407) | -0.35  (p=0.932) | -0.6  (p=0.104)* | -0.52  (p=0.058)* | 0.07  (p=0.991) | -0.12  (p=0.978) | 0.07  (p=0.936) | 0.32  (p=0.438) | -0.18  (p>0.999) | 0.5  (p=0.042) |
| Elovl3 (n-9) (b) | 0.16  (p=0.96) | 0.2  (p=0.913) | -0.05  (p=0.959) | 0.35  (p=0.407) | -0.22  (p=0.932) | 0.31  (p=0.522) | 0.09  (p=0.55) | -0.13  (p=0.991) | -0.04  (p>0.999) | -0.38  (p=0.437) | -0.41  (p=0.415) | -0.17  (p>0.999) | -0.51  (p=0.042)* |
| Elovl3 (n-9) (c) | -0.02  (p>0.999) | -0.16  (p=0.913) | -0.1  (p=0.959) | -0.38  (p=0.407) | 0.19  (p=0.932) | -0.45  (p=0.342) | -0.1  (p=0.55) | 0.07  (p=0.991) | 0.03  (p>0.999) | 0.4  (p=0.437) | 0.47  (p=0.415) | 0.24  (p>0.999) | 0.62  (p=0.018)* |
| Elovl5 (n-6) | 0.1  (p=0.96) | 0.0  (p>0.999) | -0.03  (p=0.959) | -0.25  (p=0.6) | -0.13  (p=0.932) | -0.15  (p=0.658) | 0.06  (p=0.55) | -0.13  (p=0.991) | -0.16  (p=0.978) | -0.12  (p=0.853) | 0.24  (p=0.507) | -0.03  (p>0.999) | -0.04  (p=0.443) |
| Elovl2-5 (n-6) | -0.18  (p=0.96) | -0.13  (p=0.913) | -0.21  (p=0.816) | -0.64  (p=0.07)** | -0.4  (p=0.932) | -0.48  (p=0.324) | -0.68  (p=0.022)** | -0.18  (p=0.991) | -0.28  (p=0.978) | -0.07  (p=0.936) | 0.24  (p=0.507) | -0.04  (p>0.999) | 0.24  (p=0.289) |
| Elovl2-5 (n-3) | 0.03  p>0.999 | 0.0  (p>0.999) | 0.2  (p=0.816) | 0.04  (p=0.856) | -0.13  (p=0.932) | -0.16  (p=0.658) | -0.11  (p=0.55) | -0.14  (p=0.991) | -0.18  (p=0.978) | -0.03  (p=0.985) | 0.14  (p=0.739) | 0.43  (p=0.822) | 0.13  (p=0.373) |
| Elovl2 (n-3) | -0.12  (p=0.96) | 0.31  (p=0.913) | 0.63  (p=0.076)** | 0.17  (p=0.671) | 0.27  (p=0.932) | -0.2  (p=0.597) | 0.62  (p=0.022)** | -0.4  (p=0.644) | 0.18  (p=0.978) | -0.65  (p=0.09)* | 0.38  (p=0.415) | -0.33  (p=0.95) | -0.03  (p=0.443) |
